# Risk for development of inflammatory bowel disease under inhibition of interleukin 17: A systematic review and meta-analysis

**DOI:** 10.1371/journal.pone.0233781

**Published:** 2020-05-27

**Authors:** Johan Burisch, Wolfgang Eigner, Stefan Schreiber, Daniel Aletaha, Wolfgang Weninger, Michael Trauner, Walter Reinisch, Neeraj Narula

**Affiliations:** 1 Gastrounit, Medical Division, Hvidovre Hospital, University of Copenhagen, Hvidovre, Denmark; 2 Division of Gastroenterology and Hepatology, Department of Internal Medicine III, Medical University of Vienna, Vienna, Austria; 3 Department of Gastroenterology, Christian-Albrechts-Universität zu Kiel, Kiel, Deutschland; 4 Division of Rheumatology, Department of Internal Medicine III, Medical University of Vienna, Vienna, Austria; 5 Department of Dermatology, Medical University of Vienna, Vienna, Austria; 6 Division of Gastroenterology, Department of Medicine, Farncombe Family Digestive Health Research Institute, McMaster University, Hamilton, ON, Canada; Humanitas University, ITALY

## Abstract

**Objective:**

Cases of inflammatory bowel disease (IBD) during treatment with interleukin (IL)-17 antagonists have been reported from trials in psoriasis, psoriatic arthritis, and ankylosing spondylitis. The aim of this study was to assess the overall risk for development of IBD due to IL-17 inhibition.

**Design:**

Systematic review and meta-analysis of studies conducted 2010–2018 of treatment with IL-17 antagonists in patients with psoriasis, psoriatic arthritis, ankylosing spondylitis, and rheumatoid arthritis. We compared risk of IBD development in anti-IL-17 treated patients compared to placebo treatments. We also computed incident rates of IBD overall. A ‘worst case scenario’ defining subjects ambiguous for prevalent versus incident cases for the latter was also applied.

**Results:**

Sixty-six studies of 14,390 patients exposed to induction and 19,380 patients exposed to induction and/or maintenance treatment were included. During induction, 11 incident cases of IBD were reported, whereas 33 cases were diagnosed during the entire treatment period. There was no difference in the pooled risk of new-onset IBD during induction studies for both the best-case [risk difference (RD) 0.0001 (95% CI: -0.0011, 0.0013)] and worst-case scenario [RD 0.0008 (95% CI: -0.0005, 0.0022)]. The risk of IBD was not different from placebo when including data from maintenance and long-term extension studies [RD 0.0007 (95% CI: -0.0023, 0.0036) and RD 0.0022 (95% CI: -0.0010, 0.0055), respectively].

**Conclusions:**

The risk for development of IBD in patients treated with IL-17 antagonists is not elevated. Prospective surveillance of patients treated with IL-17 antagonists with symptom and biomarker assessments is warranted to assess for onset of IBD in these patients.

## Introduction

The inflammatory bowel diseases (IBD), Crohn’s disease (CD) and ulcerative colitis (UC), are chronic inflammatory conditions which can affect various segments of the gastrointestinal tract and the colon only, respectively. Typical symptoms include diarrhea, abdominal pain and rectal bleeding, as well as development of stenoses, abscesses and fistulas in case of CD.

IBD manifests in genetically susceptible patients, potentially triggered by environmental factors and/or perturbations of the gut microbiota leading to a dysregulated mucosal immune system and development of chronic intestinal inflammation [1, 2]. In genome-wide association studies, several genetic loci were identified in patients with IBD overlapping with other immune mediated inflammatory diseases (IMIDs) such as chronic plaque psoriasis and ankylosing spondylitis [3]. Patients with psoriasis and psoriatic arthritis are more likely to develop IBD [4, 5] and there is an increased risk of developing CD in patients with ankylosing spondylitis [6].

The interleukin-17 family cytokines (IL-17A to IL-17F) that signal via several IL-17 receptors (IL-17R A to E) [7, 8] are strong inducers of inflammation contributing to tissue destruction in IMIDs. Secukinumab (SEC) and Ixekizumab (IXE), both monoclonal IgG4 antibodies directed against the IL-17A, as well as brodalumab (BRO), a monoclonal antibody directed its receptor, have been successfully used for treating various autoimmune mediated disorders such as chronic plaque psoriasis (SEC, IXE, BRO), psoriatic arthritis (SEC), and ankylosing spondylitis (SEC) [8–12].

Notably, inhibition of IL-17A has been shown to worsen colitis in mouse models [13, 14] and blocking of IL-17A and IL-17RA with the monoclonal antibodies SEC and BRO, respectively, in patients with CD not only failed efficacy, but appeared to worsen disease activity [15, 16]. The risk of IBD in patients with IMIDs treated with targeted IL-17 inhibition has so far been investigated only for specific treatments or specific IMIDs [17–19], whereas analyses combining several drugs across multiple IMIDs are lacking.

In order to expand the picture on the potential induction of IBD beyond the observed increased background risk in IMIDs, we conducted an in-depth systematic review and meta-analysis of studies with three different IL-17 inhibitors (SEC, IXE, BRO) in patients with chronic plaque psoriasis, psoriatic arthritis, ankylosing spondylitis and rheumatoid arthritis to determine whether incidence of IBD is elevated in patients treated with IL-17 antagonists compared to those treated with placebo.

## Materials and methods

Systematic review and meta-analysis were conducted according to the Preferred Reporting Items for Systematic Reviews and Meta-Analyses for Protocols. The review protocol was registered on PROSPERO (registration no. CRD42018109276).

### Search strategy and selection criteria

A systematic literature search on PubMed, EMBASE and clinicaltrials.gov using the Medical Subject Headings (MeSH) terms “Secukinumab”, “Ixekizumab”, “Brodalumab”, and “psoriasis”, “psoriatic arthritis”, “ankylosing spondylitis”, or “rheumatoid arthritis” was conducted with a cut-off date of August 31^st^ 2018 in order to identify studies with IL-17 inhibitors in patients with IMIDs reporting cases with IBD, S1 Fig. All relevant studies were selected by a single reviewer (WE) and discussed with two other authors (JB, WR). Our inclusion criteria were randomized controlled trials and open label extension studies in immune mediated diseases where treatment of anti IL-17 therapies was used with or without a comparison agent. Our outcome of interest was cases with new onset IBD (incident cases) or disease deterioration of preexisting IBD. Cases were summarized as CD and UC and finally combined as total cases of IBD. Studies including overlapping data, reviews/meta-analyses and case reports as well as abstracts were excluded. References of reviews and meta-analyses were reviewed to secure that no relevant studies have been missed in the previous search.

### Data extraction and grouping

For data extraction the original publications including supplementary material, if available, were reviewed. Data of interest comprised information on the publication itself (publication name, first author, study title, NCT, year of publication, indication, investigated drug, aim), study characteristics (number of patients included, observation period, type of study e.g. randomized controlled trial, treatment regimens and randomization) and reports of adverse events of special interest (CD, UC; relapse of pre-existing disease or new diagnosis). For unpublished studies identified through clinicaltrials.gov, we retrieved data from the European Medical Agency (EMA).

If no case of new-onset IBD was reported in a publication, we assumed that there was no such event. In some studies, detailed specifications into CD versus UC and/or new-onset cases (incident cases) versus subjects with flares of preexisting disease (prevalent cases) were not provided. Therefore, two different scenarios were assumed in the analysis process: a ‘worst case scenario’, for which all cases unclear for incident versus prevalent cases were considered new diagnoses, and a ‘best case scenario’, for which all unclear cases were considered as prevalent ones.

### Data synthesis and statistical analysis

In placebo-controlled studies, we analyzed the risk of new onset IBD in patients treated with IL-17 inhibitors compared to patients receiving placebo using the Mantel-Haenszel risk difference in a random-effects model.

Incidence rates (IRs) were calculated for all studies as the number of outcomes per 1,000 patient-years with 95% confidence intervals. Due to the low number of events, we combined all incident cases of CD and UC to incident IBD cases. As the individual treatment duration of patients was not available in all included studies, the number of person years was calculated by multiplying the number of patients with the treatment duration for each study. In studies in which no cases of IBD were reported, a correction of 0.05 was added to all columns. This would allow for inclusion of studies with zero total events, which is generally recommended when conducting meta-analyses where risk difference is the primary effect measure, as it provides a narrower range of confidence interval and decreases between-study heterogeneity [20].

IRs for all studies (both placebo-controlled and open-label extension studies) were pooled using a random-effects model. Data on incident cases from induction studies were analyzed separately to those encompassing the reported entire study period. Sub-group analyses were performed per individual drug and per indication. Data from studies comparing IL-17 inhibitors to active comparator arms was also used to calculate IRs, but we did not compare the risk of IBD in anti-IL-17 treated patients to that of active comparators given a very heterogeneous group of comparators were used in the different studies.

We evaluated the presence of heterogeneity across studies by using the I^2^ statistic, as described in the Cochrane Handbook of Systematic reviews [21]. Values of >50% were considered suggestive of substantial heterogeneity. Potential publication bias was assessed by visual inspection of funnel plots and Egger’s test [21, 22]. Statistical analyses were performed using RevMan [23] (version 5.3), the *meta* package for R [24, 25] (version 3.5.1), and Comprehensive Meta-Analysis (version 2.0).

### Assessment of study quality and risk of bias

The risk of bias was evaluated using Cochrane risk of bias tool for randomized controlled trials and the Newcastle-Ottawa scale (NOS) [26] for cohort studies to evaluate the selection, comparability and outcome of the studies. Overall quality assessment, on basis of the Grading of Recommendations Assessment, Development, and Evaluation (GRADE) criteria, was also performed.

## Results

### Search results

The literature search identified 255 citations of which 189 were excluded for duplication, retrospective case reports, unclear reporting (n = 1) [27] and providing results as exposure-adjusted incidence rates per 100 patient-years only without specifying the number of events [28]. Overall, 66 studies were eligible for review and meta-analysis (S1 Fig). S1 Table provides an overview of studies selected for meta-analysis. Among those, 43 studies qualified for meta-analysis on short-term risk assessment during induction treatment (SEC 27 studies, IXE 9 studies, BRO 7 studies), whereas the data from 48 studies were used in the meta-analysis on risk estimation for the entire treatment period (SEC 30 studies, IXE 10 studies, BRO 8 studies) with an observational period over six years (median 52 weeks, range 12–351 weeks).

There are some specifics in the reporting of cases with IBD among eligible studies deserving further detail. In the original article by Mrowietz et al. for the treatment of chronic plaque psoriasis with SEC, cases of IBD were not described as adverse events [29]. However, on clinicaltrials.gov for the same study (NCT01406938), there is one report of CD. Therefore, the latter scenario was assumed to be correct. Results for a longer exposure up to five years of treatment under SEC treatment were available in a publication by Bissonette et al. which was included in our analysis [30]. The safety and efficacy of IXE for the treatment of psoriasis was investigated by several studies within the UNCOVER program for which several cases of IBD were reported [31–33]. The publication by Gordon et al. which summarizes the results from UNCOVER 1 to 3 spanning an exposure time of 60 weeks presented a total of 11 IBD cases [31]. Two follow up studies were published from UNCOVER-3 so far, including safety data for two years and three years of treatment, respectively. Whereas from the former [32] five cases of IBD are documented, the latter mentions [33] eight IBD events (new diagnosis or relapse). As it was not clear to us whether duplicate data have been presented over those three studies, we only considered the original publication by Gordon et al. for meta-analysis [31].

For several studies, pooled data of patients receiving the study drug (after reallocation) were used, as this was the method used in the original publications to assess safety.

### Cases with IBD during induction studies

The 43 induction studies included a total of 21,893 subjects (psoriasis 14,759, psoriatic arthritis 3,403, ankylosing spondylitis 1,196, rheumatoid arthritis 2,535). Among those, 14,390 received an IL-17 blocker [SEC 7,264 (psoriasis 3,703, psoriatic arthritis 1,672, ankylosing spondylitis 801, rheumatoid arthritis 1,088), IXE 3,605 (psoriasis 2,745, psoriatic arthritis 455, rheumatoid arthritis 405), BRO 3,521 (psoriasis 3,189, psoriatic arthritis 113, rheumatoid arthritis 219) (Tables 1 and 2)]. In comparison 4,989 patients were randomized to placebo (psoriasis 2,847, psoriatic arthritis 1,062, ankylosing spondylitis 395, rheumatoid arthritis 685) and 2,514 patients to an active comparator: etanercept 1,063 patients (all psoriasis), abatacept 138 patients (all rheumatoid arthritis), fumaric acid 97 patients (all psoriasis), ustekinumab 1,115 patients (all psoriasis), and adalimumab 101 patients (all psoriatic arthritis). Treatment duration with anti-IL-17 blockers differed between studies and lasted a median 16 weeks (range 12–36 weeks). Overall, 12 cases with IBD (0.0008%) were documented from the SEC/IXE/BRO induction cohorts including either new diagnoses or relapses of preexisting CD or UC, compared to two cases (0.0004%) in patients treated with placebo and no cases in the active comparator group (Tables 1 and 2). By applying the best-case scenario, 3 out of those 12 subjects with IBD were classified as incident cases (all SEC), whereas by the worst-case analysis, 11 patients were categorized as incident IBD cases (7 SEC, 4 IXE).

**Table 1:** Overview on different therapies and occurrence of Crohn’s disease, ulcerative colitis, and inflammatory bowel disease for worst case and best case scenarios.

	Crohn’s disease					Ulcerative Colitis					Inflammatory bowel disease				
Short-term period	New diagnosis		Relapse		Total	New diagnosis		Relapse		Total	New diagnosis		Relapse		Total
	Worst Case	Best case	Worst Case	Best case		Worst Case	Best Case	Worst Case	Best case		Worst Case	Best case	Worst Case	Best case	
Secukinumab (n = 7264)	5	2	1	4	6	2	1	0	1	2	7	3	1	5	8
Placebo (n = 2685)	1	1	0	0	1	0	0	0	0	0	2	1	0	1	2*
Etanercept (n = 323)	0	0	0	0	0	0	0	0	0	0	0	0	0	0	0
Abatacept (n = 138)	0	0	0	0	0	0	0	0	0	0	0	0	0	0	0
Fumaric acid (n = 97)	0	0	0	0	0	0	0	0	0	0	0	0	0	0	0
Ustekinumab (n = 336)	0	0	0	0	0	0	0	0	0	0	0	0	0	0	0
Ixekizumab (n = 3605)	2	0	0	2	2	2	0	0	2	2	4	0	0	4	4
Placebo (n = 1256)	0	0	0	0	0	0	0	0	0	0	0	0	0	0	0
Etanercept (n = 740)	0	0	0	0	0	0	0	0	0	0	0	0	0	0	0
Adalimumab (n = 101)	0	0	0	0	0	0	0	0	0	0	0	0	0	0	0
Ustekinumab (n = 166)	0	0	0	0	0	0	0	0	0	0	0	0	0	0	0
Brodalumab (n = 3521)	0	0	0	0	0	0	0	0	0	0	0	0	0	0	0
Placebo (n = 1048)	0	0	0	0	0	0	0	0	0	0	0	0	0	0	0
Ustekinumab (n = 613)	0	0	0	0	0	0	0	0	0	0	0	0	0	0	0
**Entire treatment period**															
Secukinumab (n = 8372)	13	11	4	6	17	9	6	2	5	11	23	17	6	12	29*
Placebo (n = 1080)	2	2	0	0	2	0	0	0	0	0	2	2	0	0	2
Etanercept (n = 323)	0	0	0	0	0	0	0	0	0	0	0	0	0	0	0
Abatacept (n = 137)	0	0	0	0	0	0	0	0	0	0	0	0	0	0	0
Fumaric acid (n = 97)	0	0	0	0	0	0	0	0	0	0	0	0	0	0	0
Ustekinumab (n = 336)	0	0	0	0	0	0	0	0	0	0	0	0	0	0	0
Ixekizumab (n = 6481)	0	0	4	4	4	3	3	4	4	7	9	3	8	14	17^†^
Placebo (n = 214)	0	0	0	0	0	0	0	0	0	0	0	0	0	0	0
Ustekinumab (n = 166)	0	0	0	0	0	0	0	0	0	0	0	0	0	0	0
Brodalumab (n = 4527)	1	1	0	0	1	0	0	0	0	0	1	1	0	0	1
Placebo (n = 111)	0	0	0	0	0	0	0	0	0	0	0	0	0	0	0
Ustekinumab (n = 613)	0	0	0	0	0	0	0	0	0	0	0	0	0	0	0

* in one patient IBD was not further subcategorized

†in one study six cases of IBD were not further subcategorized.

**Table 2:** Overview on anti-IL 17 therapies, placebo and active controls on occurrence of Crohn’s disease, ulcerative colitis, and inflammatory bowel disease for worst case and best case scenarios for short-term period and entire treatment.

	Crohn’s disease					Ulcerative Colitis					Inflammatory bowel disease				
Short-term period	new diagnosis		relapse		total	new diagnosis		relapse		total	new diagnosis		relapse		total
	Worst case	Best case	Worst case	Best case		Worst Case	Best case	Worst case	Best case		Worst case	Best case	Worst case	Best case	
All anti IL-17 (n = 14390)	7	2	1	6	8	4	1	0	3	4	11	3	1	9	12
Placebo (n = 4989)	1	1	0	0	1	0	0	0	0	0	2	1	0	1	2*
Active controls (n = 2514)	0	0	0	0	0	0	0	0	0	0	0	0	0	0	0
**Entire treatment**															
All anti IL-17 (n = 19380)	14	12	8	10	22	12	9	6	9	18	33	21	14	26	47^†^
Placebo (n = 1405)	2	2	0	0	2	0	0	0	0	0	2	2	0	0	2
Active controls (n = 1672)	0	0	0	0	0	0	0	0	0	0	0	0	0	0	0

* in one patient IBD was not further subcategorized

†seven cases of IBD were not further subcategorized.

### Cases with IBD during the entire treatment period

For the assessment of subjects with IBD during the entire treatment period with anti-IL-17 blockers, data on 22,320 patients was available with a total of 23,652 patients-years of follow-up. Among those, 19,380 patients were treated with either SEC (psoriasis 3,672, psoriatic arthritis 2,072, ankylosing spondylitis 1,164, rheumatoid arthritis 1,464), IXE (psoriasis 5,385, psoriatic arthritis 631, rheumatoid arthritis 465) or BRO (psoriasis 4,152, psoriatic arthritis 156, rheumatoid arthritis 219; Tables 1 and 2). The number of patients exposed to placebo and active comparators were 1,405 (psoriasis 840, psoriatic arthritis 464, ankylosing spondylitis 6, rheumatoid arthritis 95) and 1,672 (etanercept 323 patients with psoriasis, abatacept 137 patients with rheumatoid arthritis, fumaric acid 97 patients with psoriasis, ustekinumab 1,115 patients with psoriasis), respectively. All available data on patients with active treatment were included with a median follow up time of 52 weeks (range 12–351 weeks). Overall, 47 cases of IBD (0.024%) were reported compared to two reports exposed to placebo (0.14%) and none in the active comparator groups (Tables 1 and 2). According to the best-case scenario 21 of those 47 subjects with IBD were classified as incident cases (17 SEC, 3 IXE, 1 BRO) whereas by the worst-case analysis, 33 patients were categorized as incident IBD cases (23 SEC, 9 IXE, 1 BRO).

### Meta-analysis

**Fig 1:**
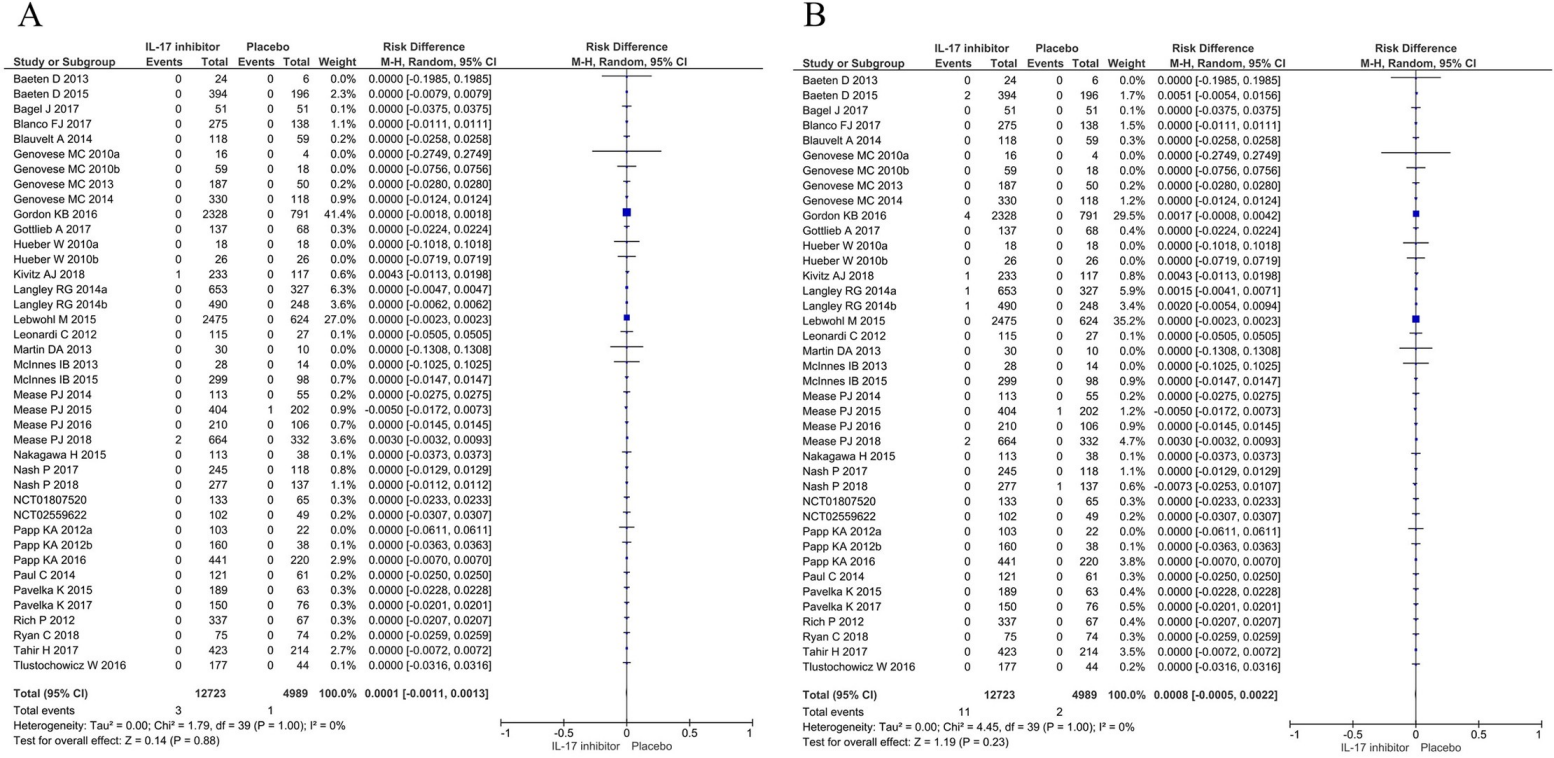
Meta-analysis of risk differences in induction studies with IL-17 inhibitors assuming a best case (A) or worst case (B) scenario.

**Fig 2:**
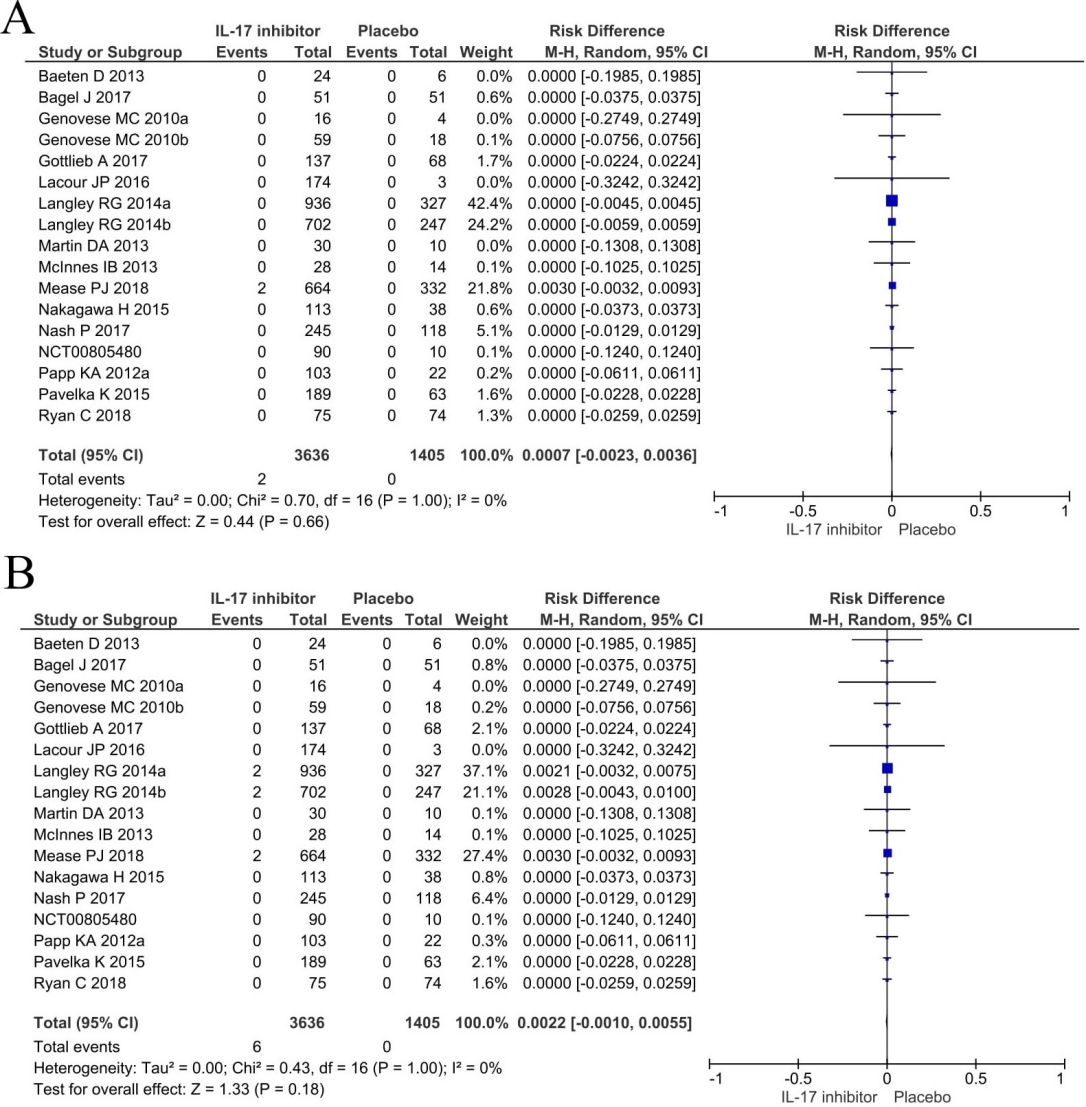
Meta-analysis of risk differences in studies of the entire treatment period with IL-17 inhibitors assuming a best case (A) or worst case (B) scenario.

**Fig 3:**
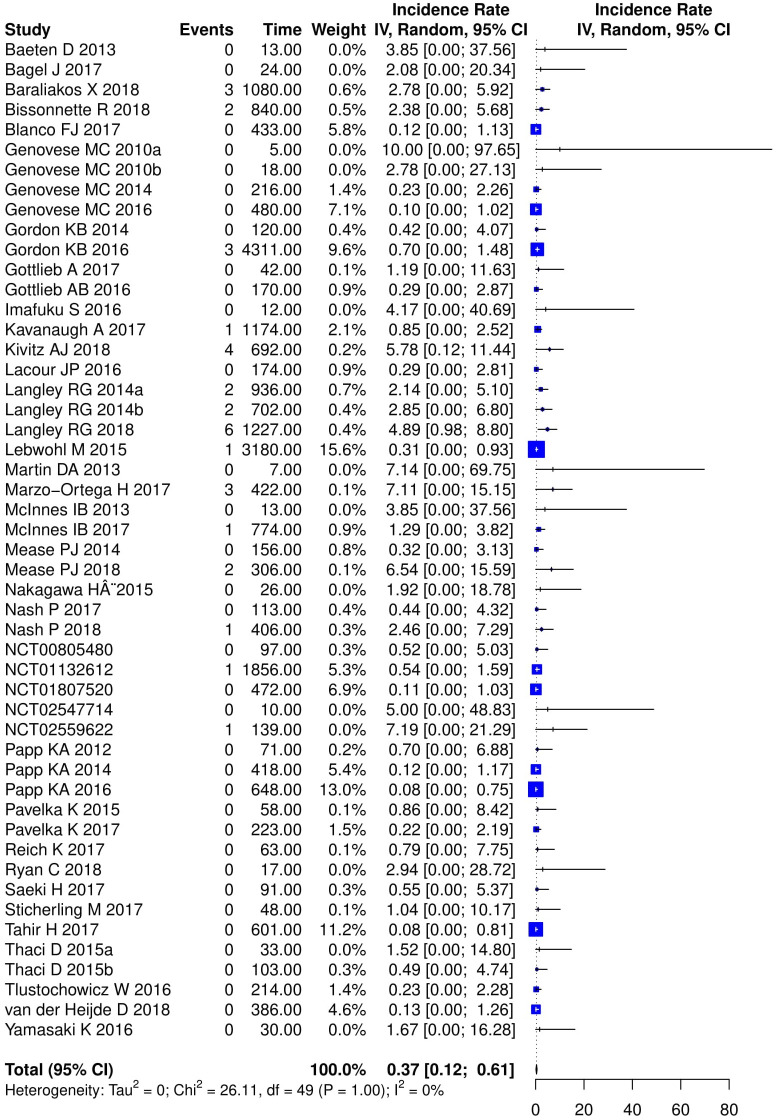
Meta-analysis of incidence rates of new-onset inflammatory bowel disease in studies of the entire treatment period with IL-17 inhibitors assuming a worst case scenario.

Comparing patients receiving any IL-17 inhibitor to placebo, there was no difference in the pooled risk of new-onset IBD in the induction studies for both the best-case [RD 0.0001 (95% CI: -0.0011, 0.0013)] and worst-case scenario [RD 0.0008 (95% CI: -0.0005, 0.0022)]. The same was the case for studies of the entire treatment period [RD 0.0007 (95% CI: -0.0023, 0.0036) and RD 0.0022 (95% CI: -0.0010, 0.0055, respectively)]. Pooled RD are summarized in Figs 1 and 2. These findings proved to remain consistent across all conducted sub-analyses: there was no difference in the pooled risk per individual drug (S2–S5 Figs) or indication (S6–S9 Figs). We did not find any heterogeneity among all RD estimates (I^2^ = 0 for all estimates in Figs 1–3; S2–S9 Figs, S11–S18 Figs). There was no evidence of publication bias as assessed by funnel plots (S10 Fig) and Egger’s test (all p-values > 0.05 for induction studies and all studies comparing IL-17 and placebo treated patients in best-case and worst-case scenarios).

We also assessed the incidence rates of IBD in the group of patients treated with IL-17 inhibitors. After combining new-onset IBD cases across all studies by the less conservative worst-case scenario we observed a pooled incidence rate of 0.37 per 1,000 patient-years (95% CI: 0.12, 0.61) for the entire treatment period (Fig 3). In the subgroup analysis, patients with ankylosing spondylitis had the highest incidence rates [IR: 2.48 per 1,000 patient-years (95% CI: 0.00; 5.03)] but the rates were not significantly different than the IRs in other indications (S11 Fig). Pooled incidence rates among the different IL-17 inhibitors were similar (S12 Fig). The best-case scenario approach did not produce significant pooled incidence rates (S13–S15 Figs), neither did the analysis of induction studies only in the worst-case scenario (S16–S18 Figs).

Individual study quality assessments are provided in S2 Table. The overall quality assessment was based on the Grading of Recommendations, Assessment, Development, and Evaluation (GRADE) criteria. Randomized trials start with a high level of evidence and this was further down-categorized to a moderate level of evidence as occurrence of IBD was not predefined as an outcome of interest in most trials and cases were reported spontaneously. The same issues were noted in observational studies. Furthermore, most open label trials only assessed safety of a single treatment arm without a control group. The starting level of evidence for observational studies according to GRADE criteria was low and was adjusted to very low for the reasons mentioned above.

## Discussion

This meta-analysis of over 19,000 patients treated with SEC, IXE and BRO for psoriasis, psoriatic arthritis, ankylosing spondylitis and rheumatoid arthritis spanning an exposure for over six years, did not observe an increased risk of incident IBD in these patients. In our analysis, 47 cases of IBD [pooled incidence rate of 0.37 per 1,000 patient-years (95% CI: 0.12, 0.61) for worst case scenario were reported in patients treated with IL-17 antagonists. In comparison, although the incidence varies throughout the world, studies from North America and Europe suggest an incidence rate of IBD of up to 0.30 per 1,000 patient-years [34]. Given that the incidence is known to be higher in patients with immune-mediated diseases, the pooled incidence rate in our meta-analysis is in keeping with the baseline risk of IBD in this patient population [5, 35].

During the entire treatment period, most cases of IBD were observed in patients treated with SEC [IR: 0.45 per 1,000 patient-years (95% CI: 0.07; 0.82) for all indications, worst case scenario], and IXE [IR: 0.46 per 1,000 patient-years (95% CI: 0.00; 0.97)]. Notably, in trials assessing efficacy and safety of BRO only one event of CD was documented [12]. This finding could point to potential differences in inhibition of the cytokine itself compared to its receptor with regard to the onset of IBD. In a retrospective analysis by Schreiber et al. [19] assessing incident rates of IBD in patients treated with SEC, 7355 patients were included and 30 new cases of CD or UC were documented. In our analysis, 8372 patients were treated with SEC with a total of 23 new reported cases of IBD. Both studies have suggested no increased risk for development of IBD in patients with SEC.

Regarding the studied indications, most randomized patients were afflicted with psoriasis, while fewer patients were treated for psoriatic arthritis, ankylosing spondylitis or rheumatoid arthritis. Among these indications, most incident cases occurred in patients with ankylosing spondylitis [IR: 2.48 per 1,000 patient-years (95% CI: 0.00; 5.03)], followed by psoriasis [IR: 0.40 per 1,000 patient-years (95% CI: 0.09; 0.71)] and psoriatic arthritis [IR: 0.56 per 1,000 patient-years (95% CI: 0.00; 1.37)]. These findings suggest a potentially increased risk for occurrence of IBD under inhibition of IL-17. However, these results have to be interpreted with caution as the studies included were heterogeneous. Furthermore, an inherently increased co-aggregation of various IMIDs such as CD, UC, psoriasis and psoriatic arthritis could contribute to an increased number of IBD incident cases in the reviewed anti-IL-17/IL-17R studies [4, 5, 36]

In most trials, IBD was not pre-defined as an adverse event of interest and cases were only spontaneously reported. It is not clear if gastroenterologists were involved in case adjudication, and whether patients underwent diagnostic procedures to confirm a diagnosis of IBD. In some studies, occurrence of IBD was not specifically mentioned [37–46], potentially contributing to an under-reporting of cases. For instance, in studies evaluating safety of SEC in rheumatoid arthritis, only reports of ‘gastrointestinal disorders’ without further clarification were mentioned [40, 41]. Remarkably, in many studies, adverse events such as diarrhea and abdominal pain were reported on a frequent basis providing no further details as to whether those events were of temporary or persistent nature. Furthermore, the differential diagnostic process between CD and UC is not detailed which suggests a potential for diagnostic misclassification within IBD. Consequently, we decided to run our analysis on an aggregate level with all diagnoses of IBD.

We had to exclude some publications from our meta-analysis due to repetition and partially dissenting reporting of numbers of patients with IBD from study results of phase III trials and their long-term extensions, a circumstance that most likely only marginally impacts our findings. In some publications the number of patients with IBD at baseline remained elusive. We addressed ambiguities in the distinction between incident versus prevalent cases of IBD under IL-17 blockade by running our meta-analysis on two different scenarios: a conservative best case scenario, by which unclear cases are accounted as incident ones and a more progressive worst case scenario, which defined unclear cases as prevalent ones. Even though those analyses led to obvious shifts in diagnoses of incident cases, this did not impact our overall results. Another limitation of our study is that we did not have access to the individuals’ exposure time. We therefore were not able to explore potential relationships on duration of drug exposure and occurrence and magnitude of adverse events of special interest. Also based on our data, we cannot conclude whether these potentially IBD-inducing effects of IL-17 directed therapies are permanent or reversible upon cessation of treatment.

Finally, the results of our analysis should be interpreted with caution due to several reasons. The duration of follow-up was short in many trials and both follow-up times and incidence rates varied among trials, as did the study settings which therefore could bias the results. Furthermore, the number of studies with zero events among both the placebo and IL-17 inhibitor treated groups was high. Therefore, the power of the study is potentially limited. While the inclusion of such studies is recommended when conducting meta-analyses of risk differences [20, 47], it is methodologically challenging as several methods for performing meta-analysis become impossible due to division of zero. For the meta-analysis of risk differences, we used the Mantel-Haenszel method which incorporates evidence from zero-event studies without requiring continuity corrections, unless the number of events is zero for all studies. For the meta-analysis of incidence rates, we used a continuity correction to bypass the complications associated with zero events. This method has been shown to produce less biased results compared to ignoring zero-value results [48]. We do, however, acknowledge the potential bias in this method as arbitrarily adding a value might potentially swamp any real effects. Also, as we were not able to retrieve data on length of treatment with IL-17 inhibitors when analyzing incidence rates, we assumed that all patients were treated for the full duration of the trial. Thereby, we could have overestimated the number of available patient-years and hence underestimated incidence rates.

To date this review and meta-analysis is the most comprehensive analysis of data concerning a potential association between blocking IL-17 and occurrence of IBD. Another strength is that in this analysis we used several approaches to account for potential events of IBD, as information on new diagnosis or disease relapse was missing in several studies. In conclusion, while we did not find an increased risk for IBD after initiation of anti-IL-17 directed therapies, close monitoring of symptoms and biomarkers which may suggest IBD before and during treatment with SEC, IXE, and BRO appears reasonable. Further prospectively conducted studies investigating the occurrence of IBD in populations at risk (e.g. patients with diseases sharing IL-23 receptor gene polymorphisms, therapy with SEC or IXE) are warranted.

## Supporting information

S1 Checklist PRISMA 2009 checklist. (DOC)

S1 Fig Study selection process. (TIF)

S2 Fig Forest plot, short-term, best-case scenario, per drug. (JPG)

S3 Fig Forest plot, entire, best-case scenario, per drug. (JPG)

S4 Fig Forest plot, entire, worst-case scenario, per drug. (JPG)

S5 Fig Forest plot, short-term, worst-case scenario, per drug. (JPG)

S6 Fig Forest plot, short-term, best-case scenario, per indication. (JPG)

S7 Fig Forest plot, entire, best-case scenario, per indication. (JPG)

S8 Fig Forest plot, entire, worst-case scenario, per indication. (JPG)

S9 Fig Forest plot, short-term, worst-case scenario, per indication. (JPG)

S10 Fig Funnel plot. A) Short-term, best-case, B) short-term, wort-case, C) entire, best-case, D) entire, worst-case.
(TIFF)

S11 Fig Forest plot, entire, worst-case scenario, per drug with correction for zero-event studies. (JPG)

S12 Fig Forest plot, entire, best-case scenario with correction for zero-event studies. (JPG)

S13 Fig Forest plot, entire, best-case scenario, per drug with correction for zero-event studies. (JPG)

S14 Fig Forest plot, entire, worst-case scenario, per indication with correction for zero-event studies. (JPG)

S15 Fig Forest plot, entire, best-case scenario, per indication, per indication with correction for zero-event studies. (JPG)

S16 Fig Forest plot, short-term, worst-case scenario with correction for zero-event studies. (JPG)

S17 Fig Forest plot, short-term, worst-case scenario, per drug with correction for zero-event studies. (JPG)

S18 Fig Forest plot, short-term, worst-case scenario, per indication with correction for zero-event studies. (JPG)

S1 Table Studies included in the systematic review. (DOCX)

S2 Table Risk of bias assessment. (DOCX)

## Abbreviations

CD: Crohn’s disease
IBD: Inflammatory bowel disease
UC: Ulcerative colitis
SEX: Secukinumab
IXE: Ixekizumab
BRO: Brodalumab
