# Supplementary figures and images for: Risk for development of inflammatory bowel disease under inhibition of interleukin 17: A systematic review and meta-analysis
Source: PLoS One. 2020 May 27;15(5):e0233781. doi: 10.1371/journal.pone.0233781 (PMC7252630; doi:10.1371/journal.pone.0233781)

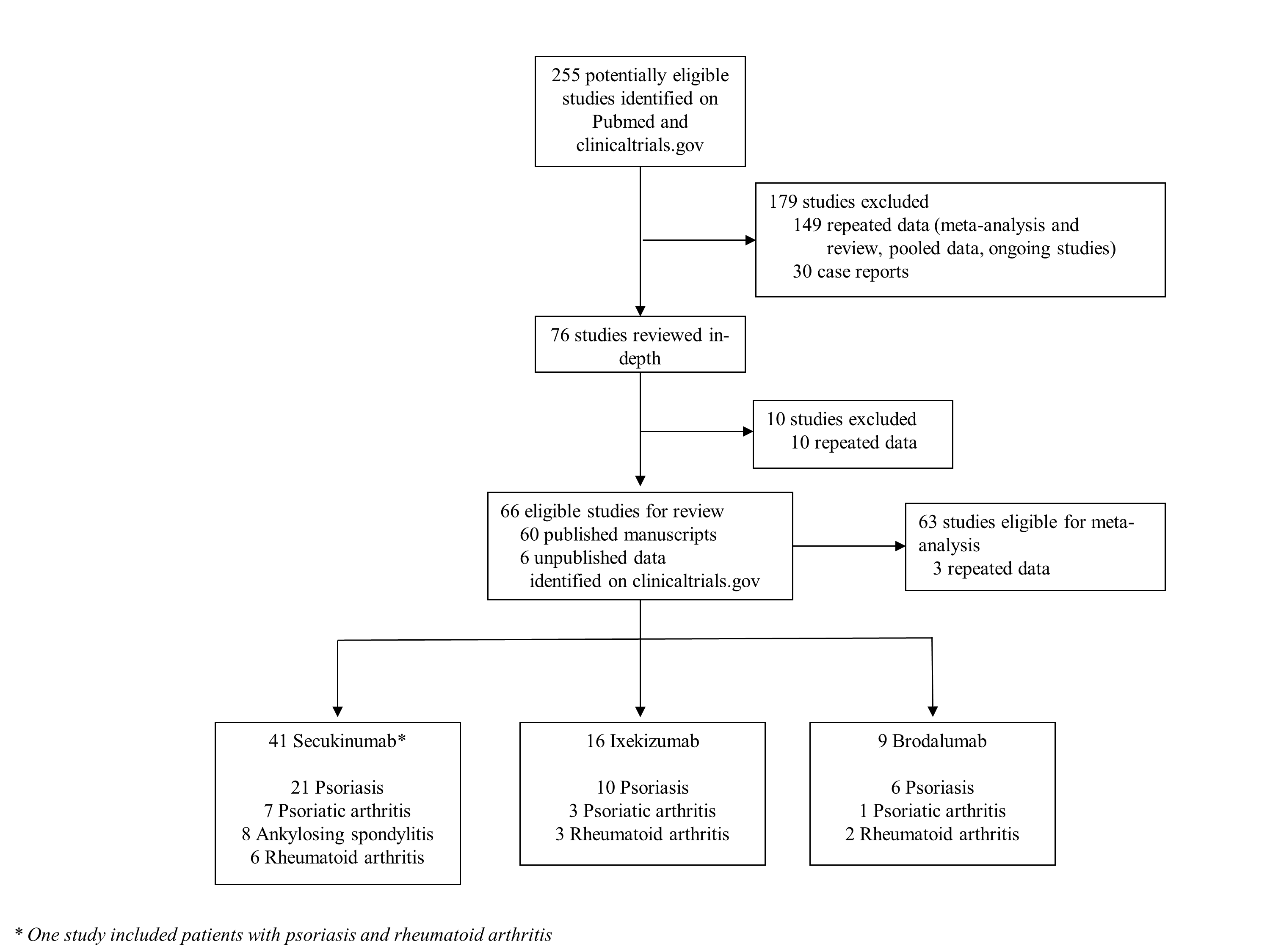

Supplement: S1 Fig — (TIF) [file pone.0233781.s002.tif]

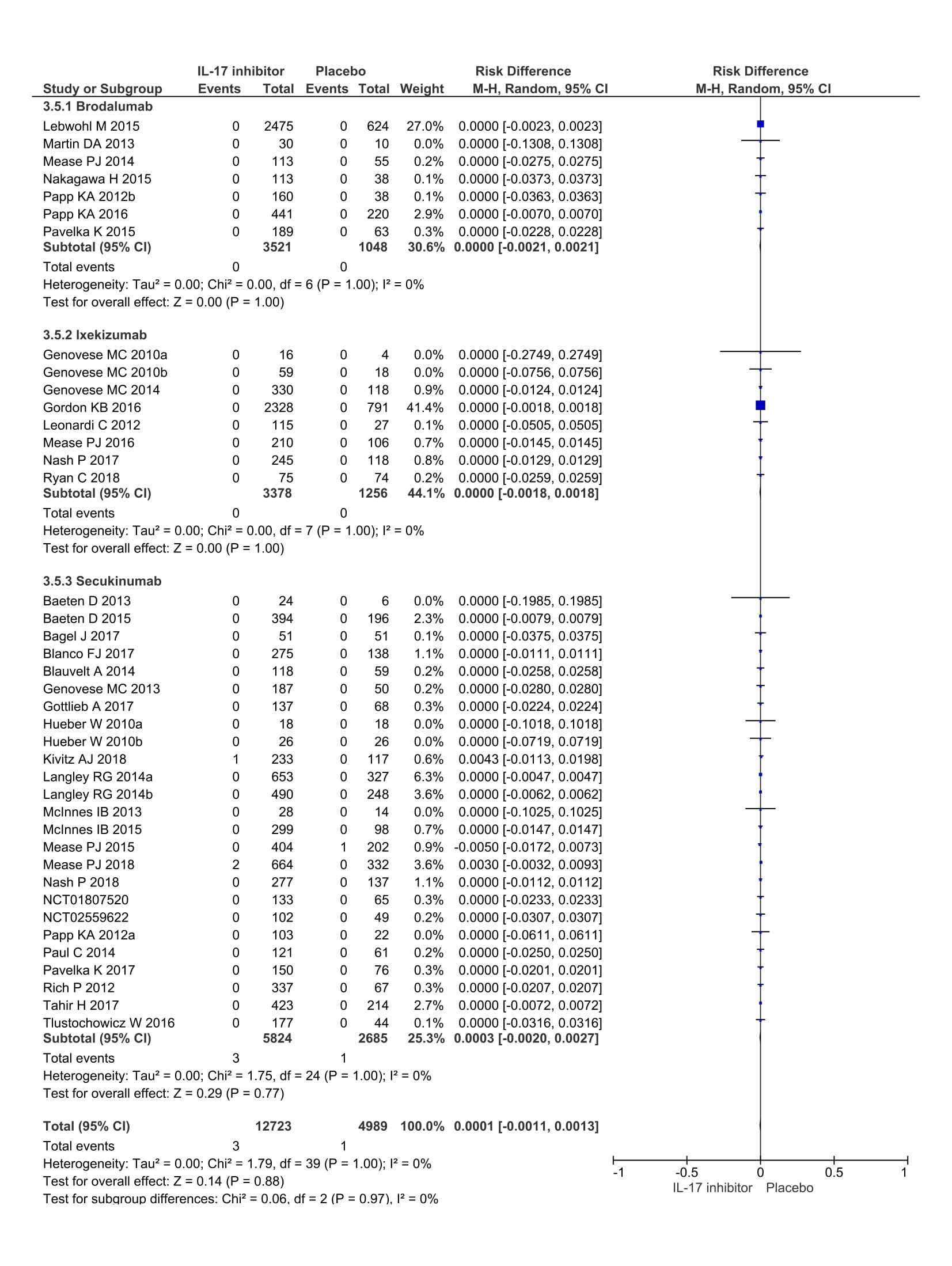

Supplement: S2 Fig — (JPG) [file pone.0233781.s003.jpg]

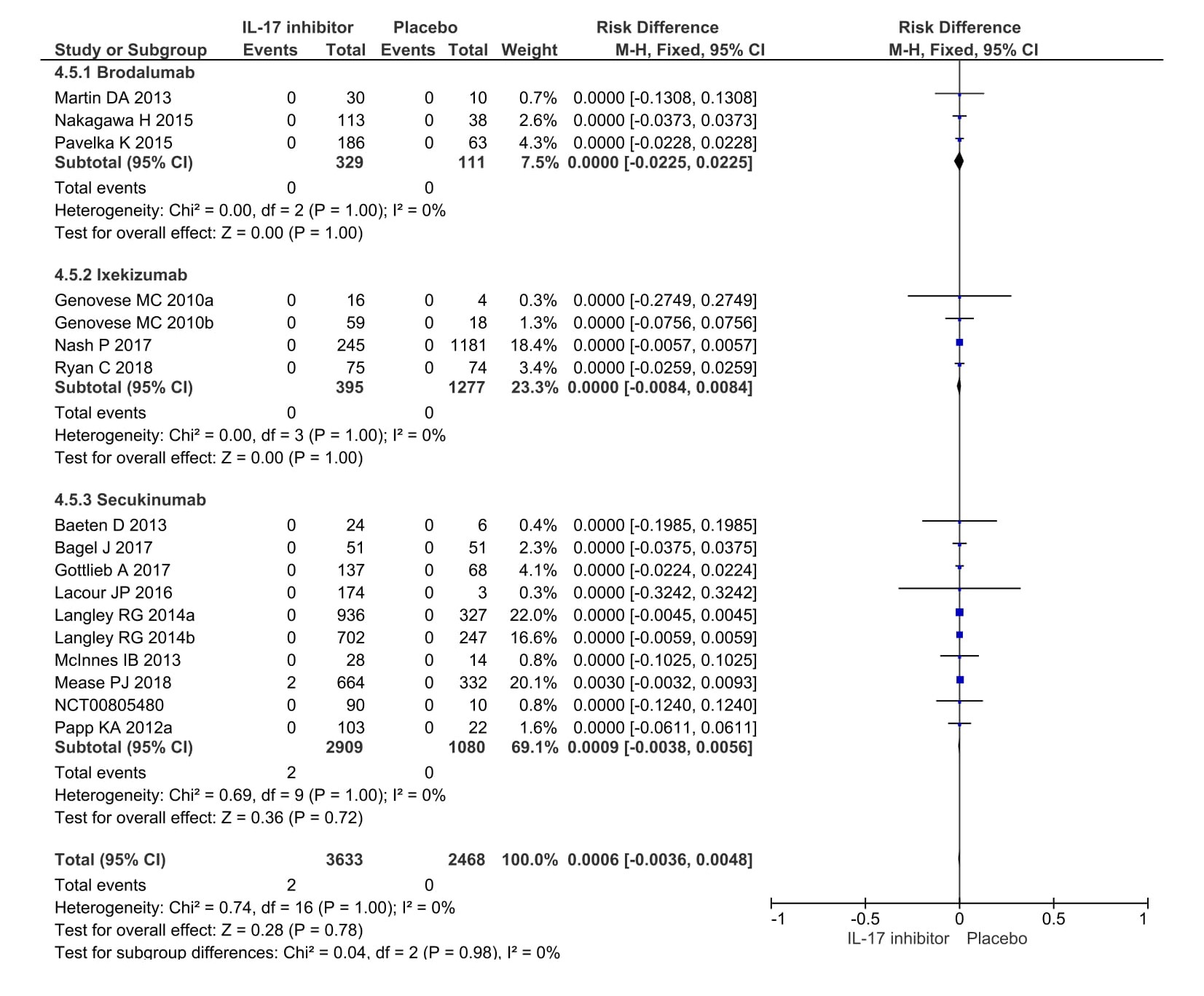

Supplement: S3 Fig — (JPG) [file pone.0233781.s004.jpg]

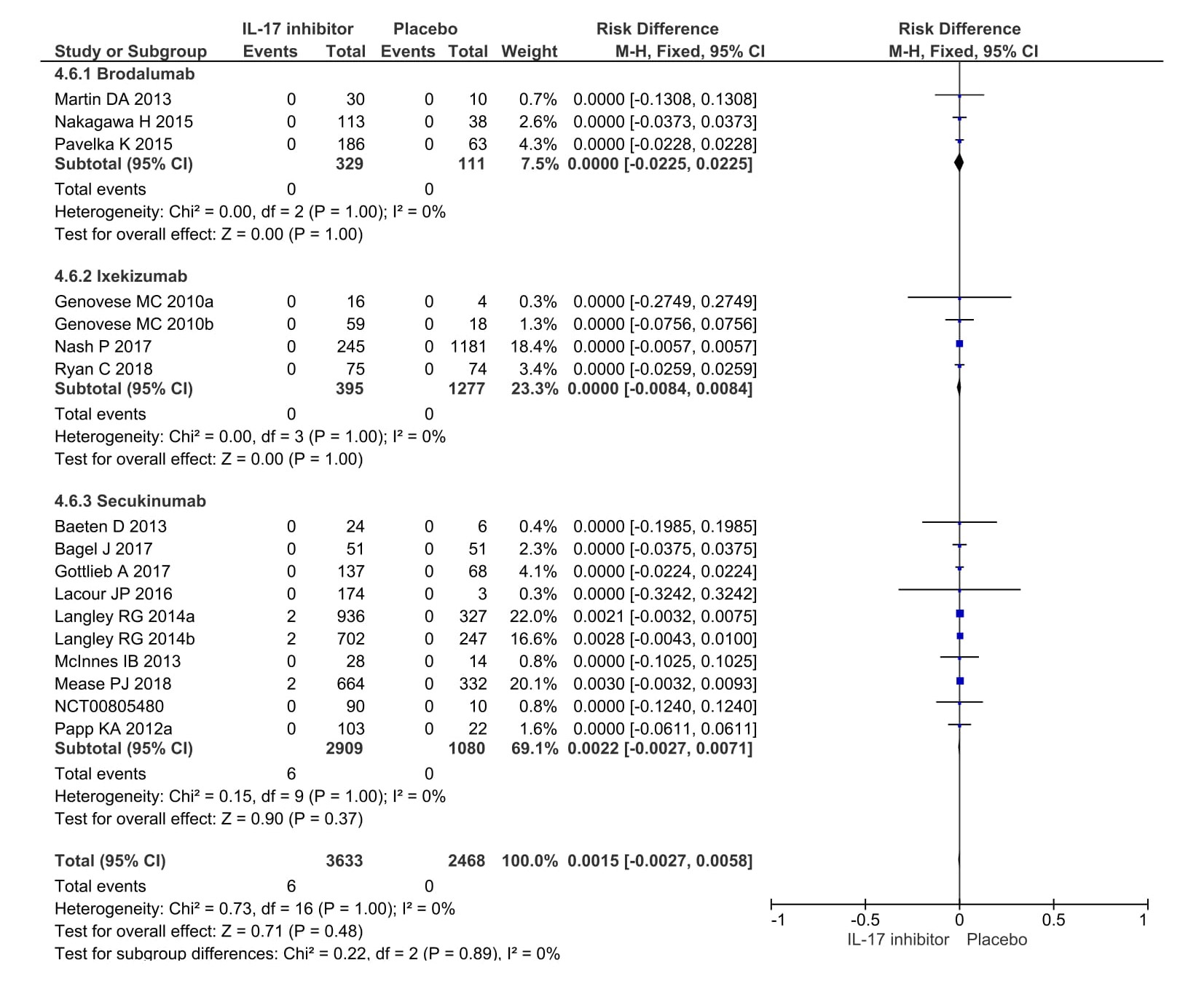

Supplement: S4 Fig — (JPG) [file pone.0233781.s005.jpg]

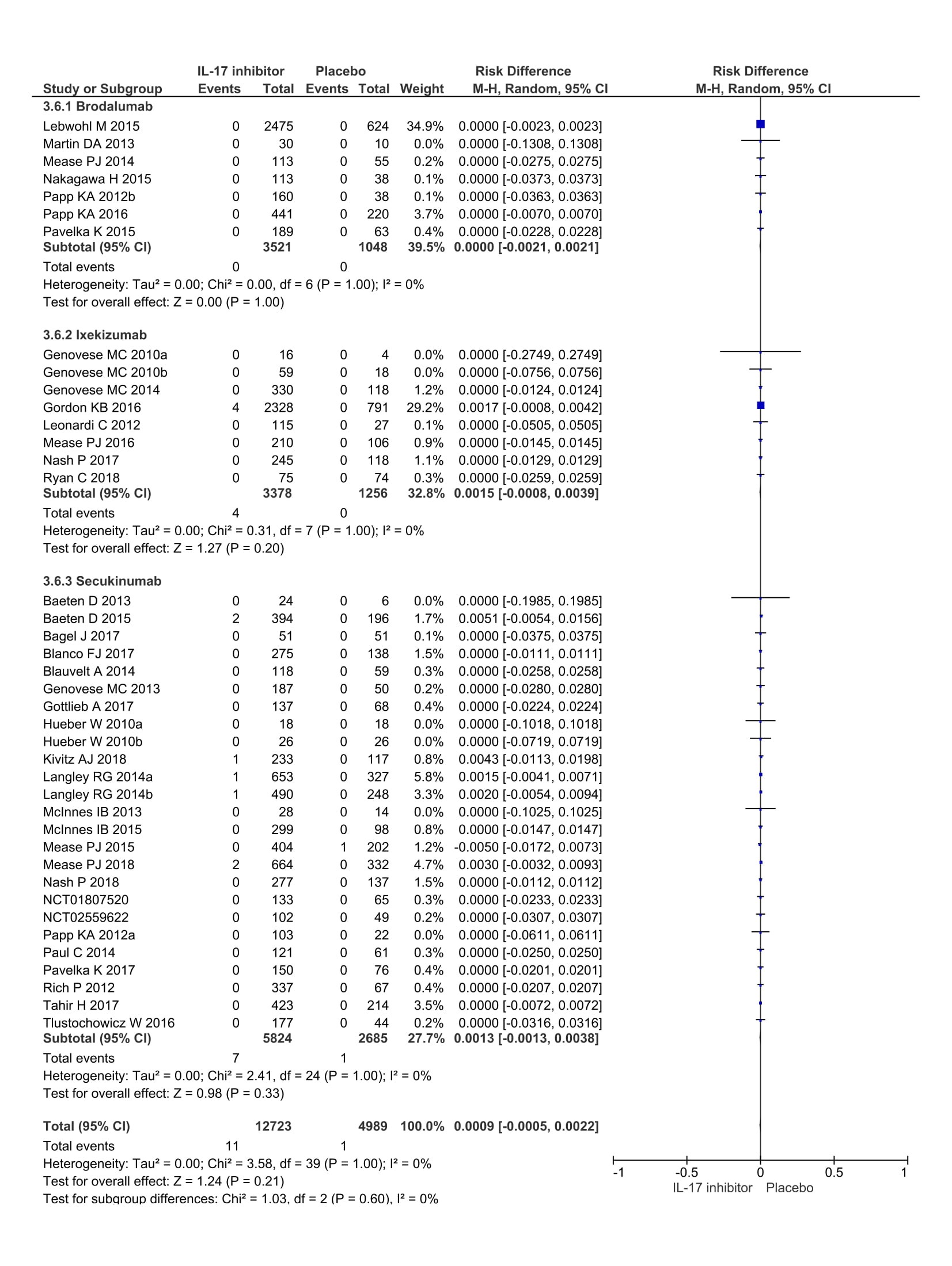

Supplement: S5 Fig — (JPG) [file pone.0233781.s006.jpg]

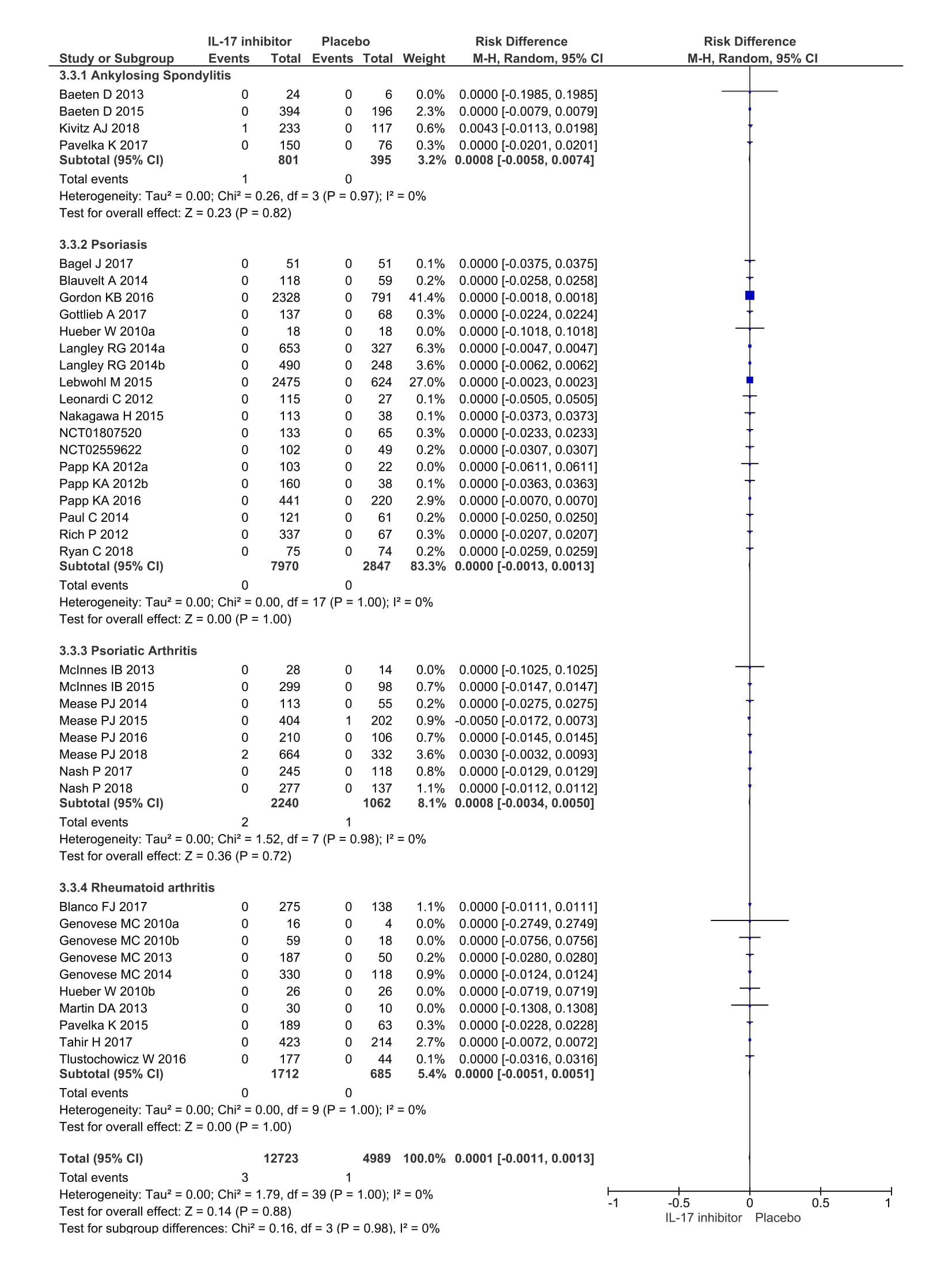

Supplement: S6 Fig — (JPG) [file pone.0233781.s007.jpg]

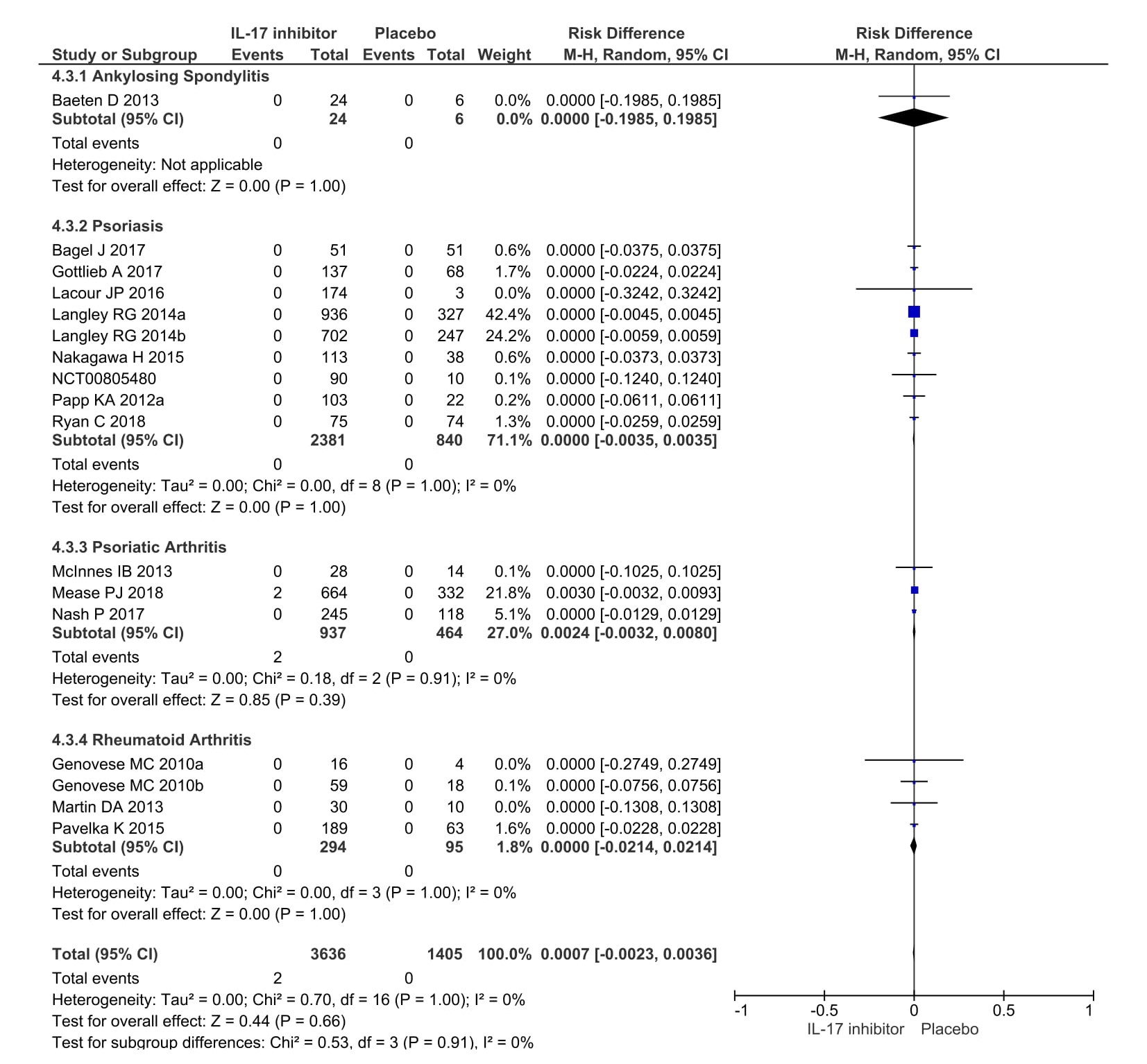

Supplement: S7 Fig — (JPG) [file pone.0233781.s008.jpg]

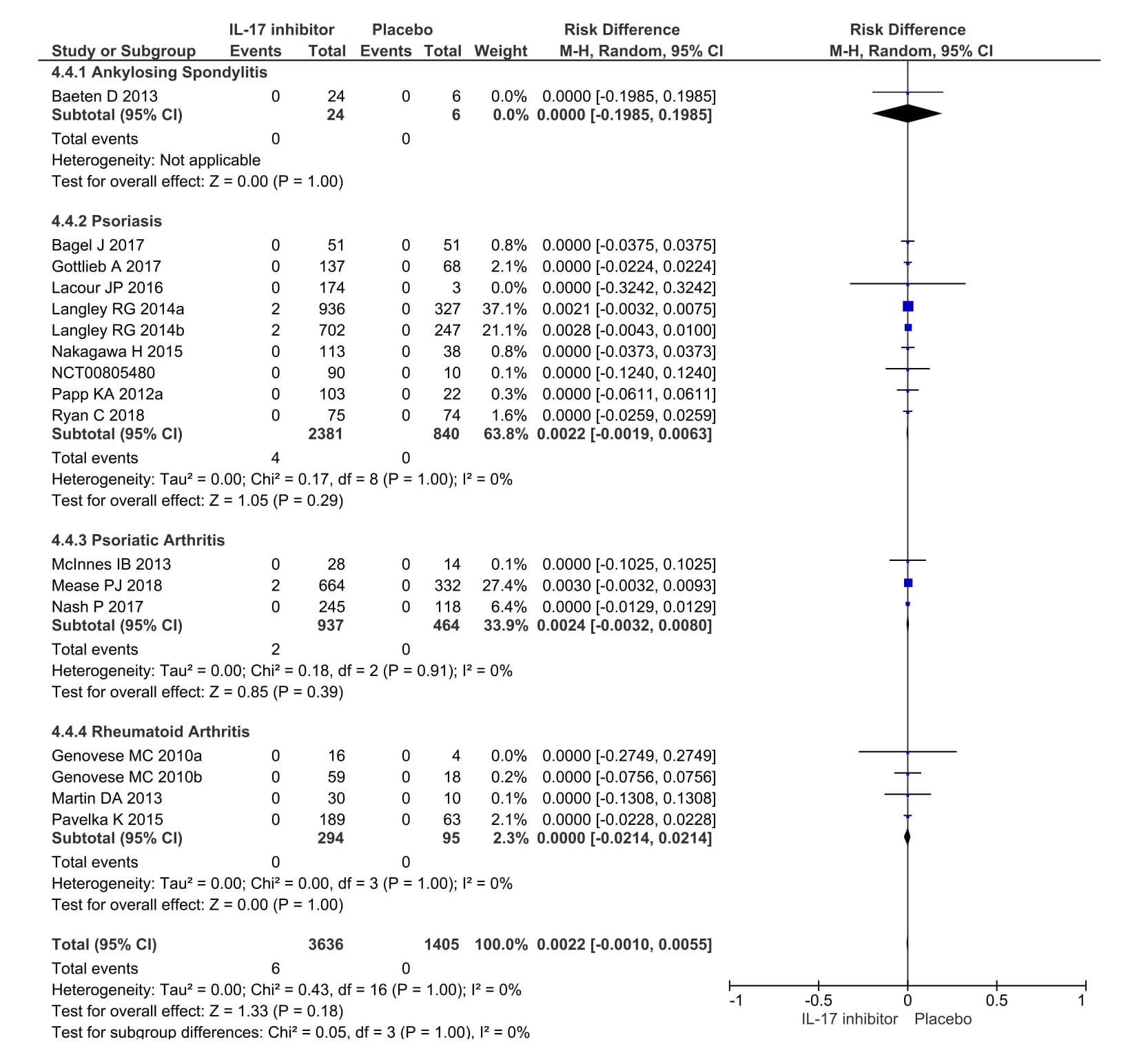

Supplement: S8 Fig — (JPG) [file pone.0233781.s009.jpg]

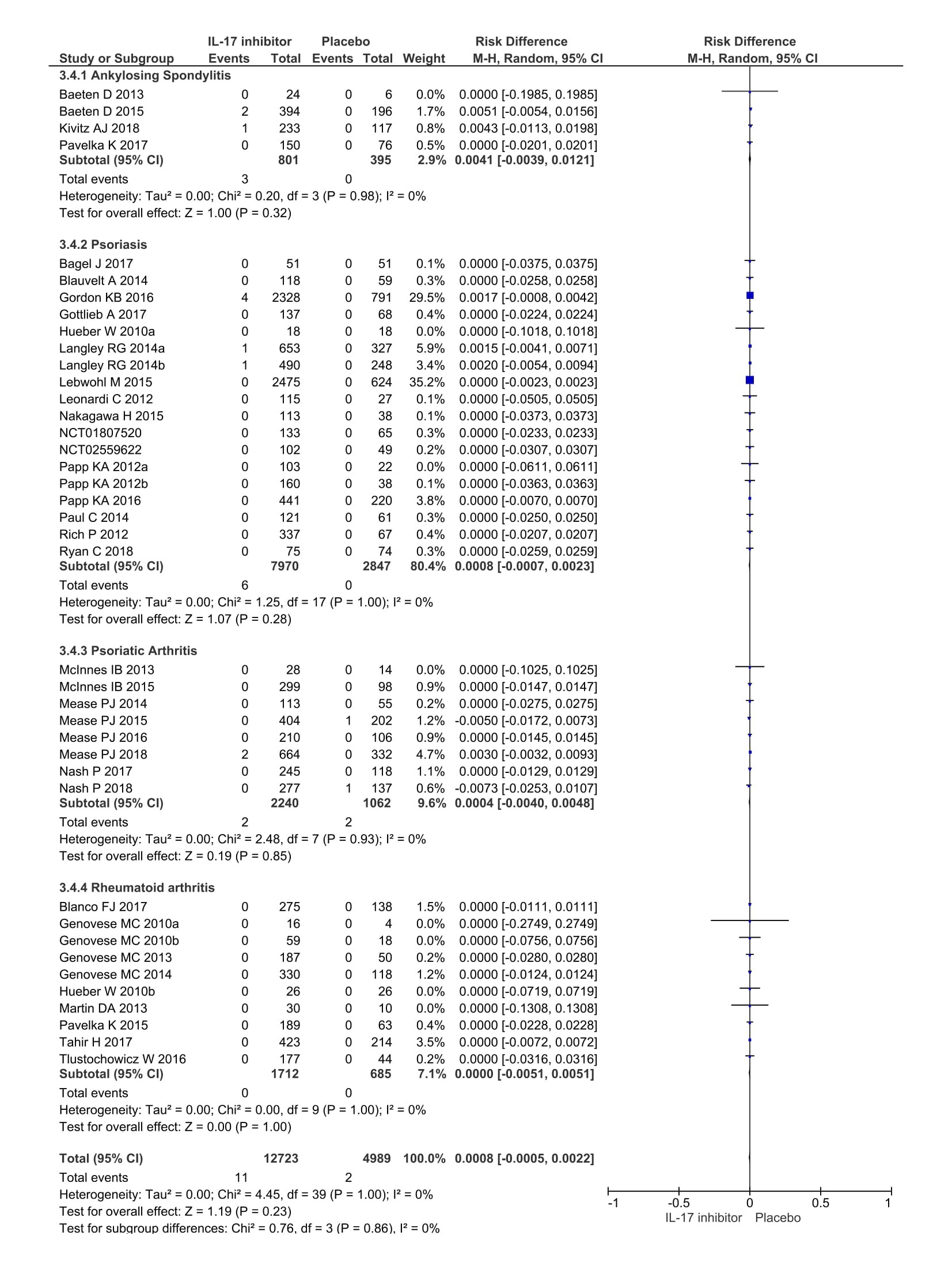

Supplement: S9 Fig — (JPG) [file pone.0233781.s010.jpg]

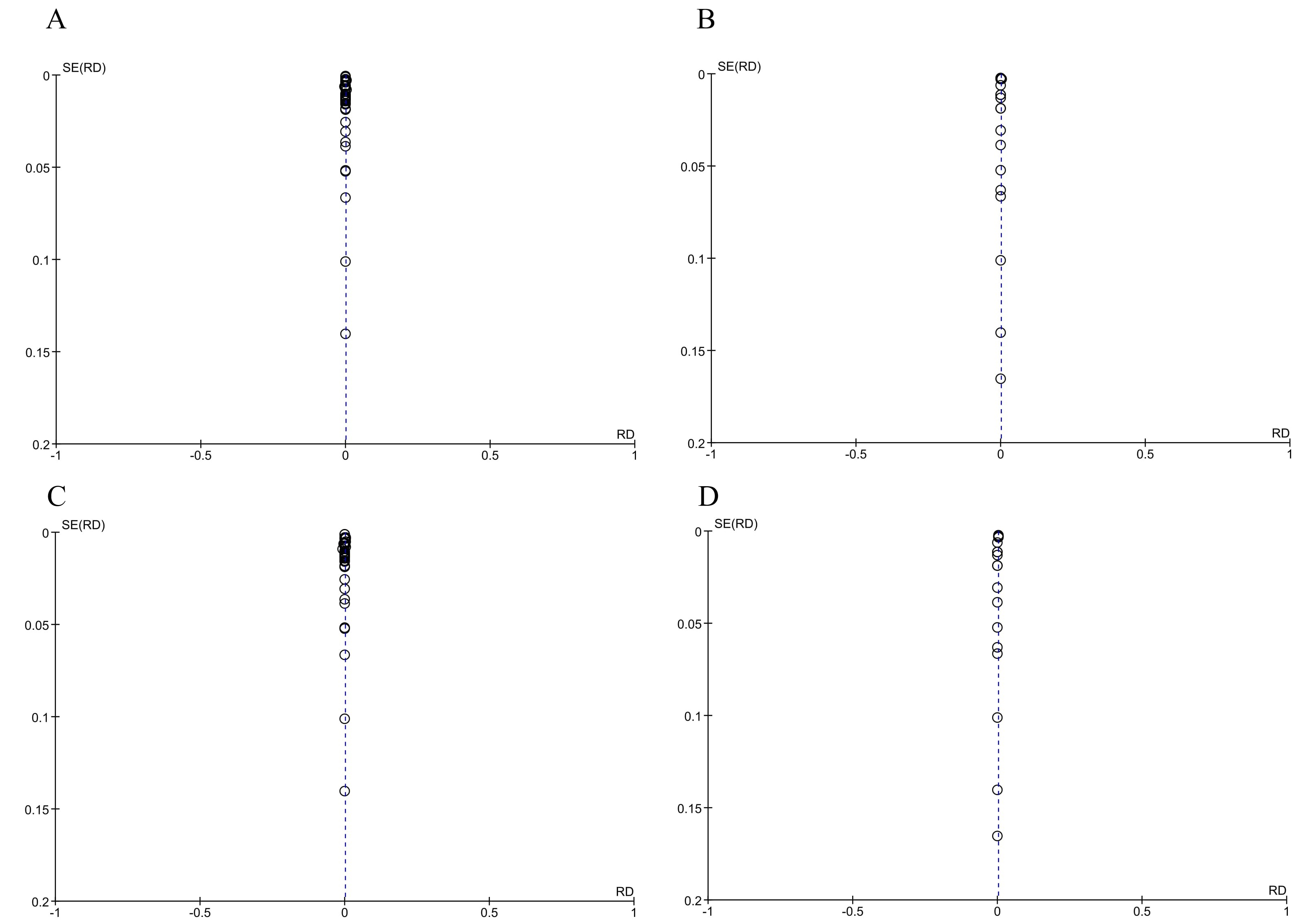

Supplement: S10 Fig — A) Short-term, best-case, B) short-term, wort-case, C) entire, best-case, D) entire, worst-case. (TIFF) [file pone.0233781.s011.tiff]

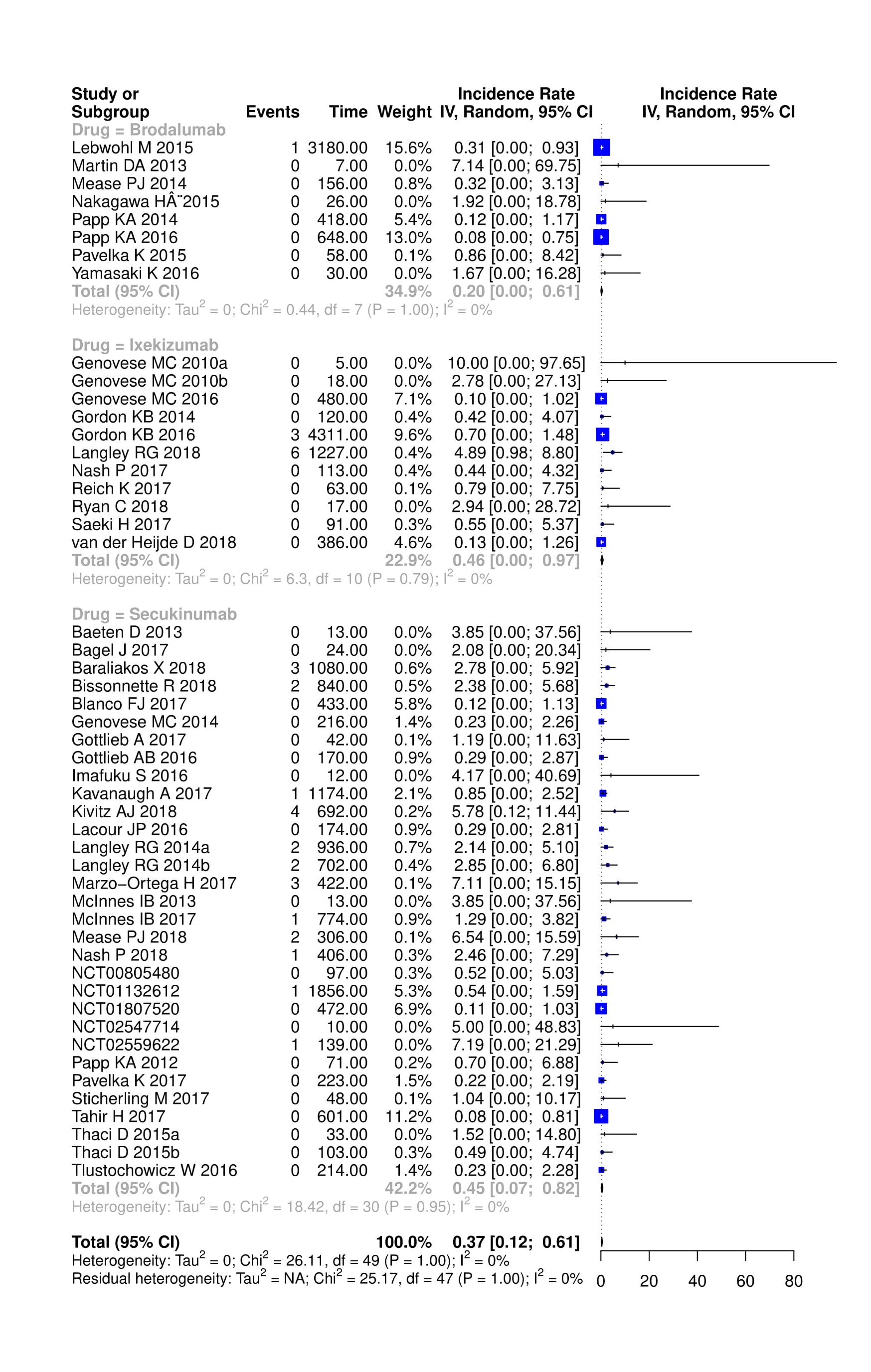

Supplement: S11 Fig — (JPG) [file pone.0233781.s012.jpg]

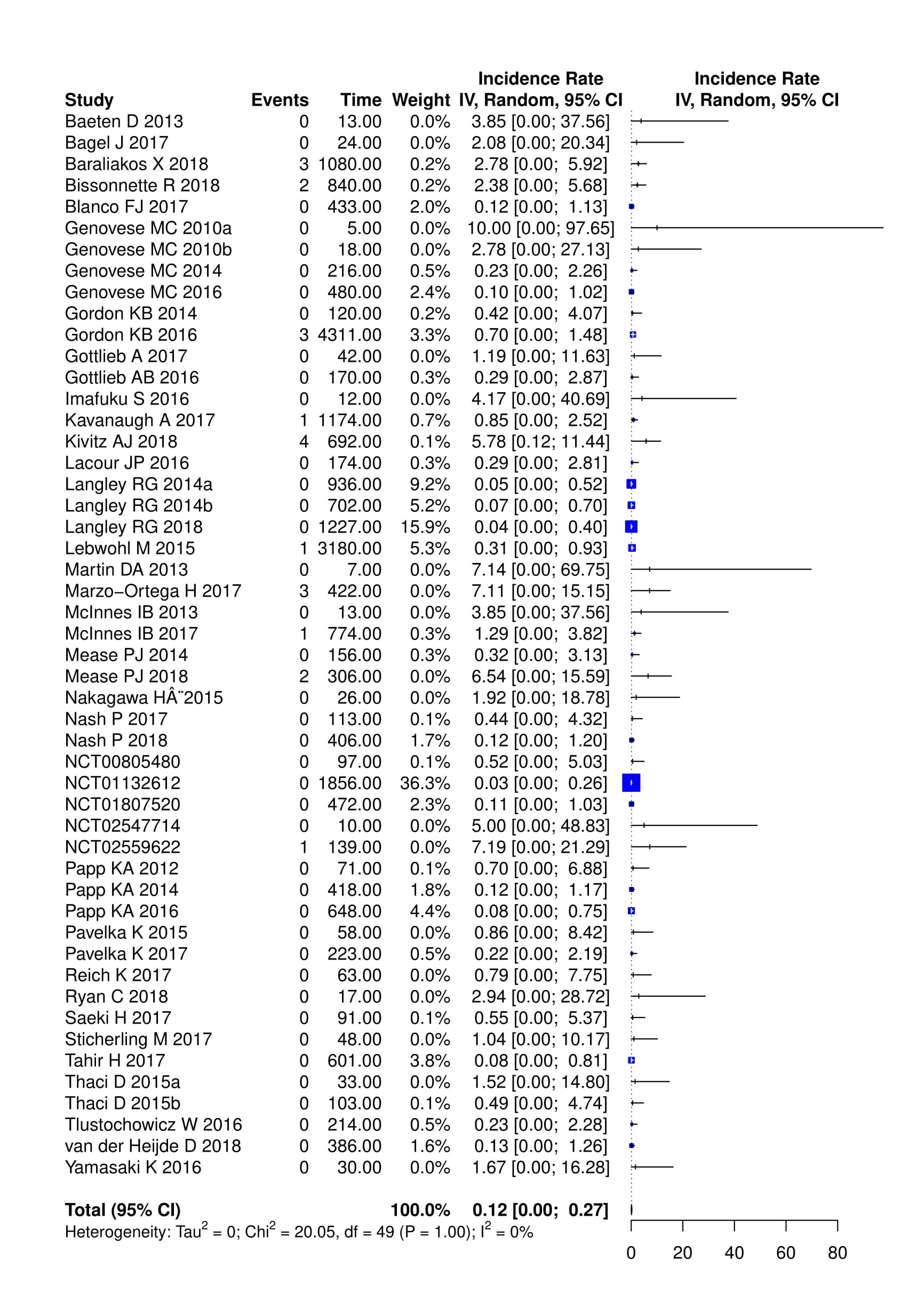

Supplement: S12 Fig — (JPG) [file pone.0233781.s013.jpg]

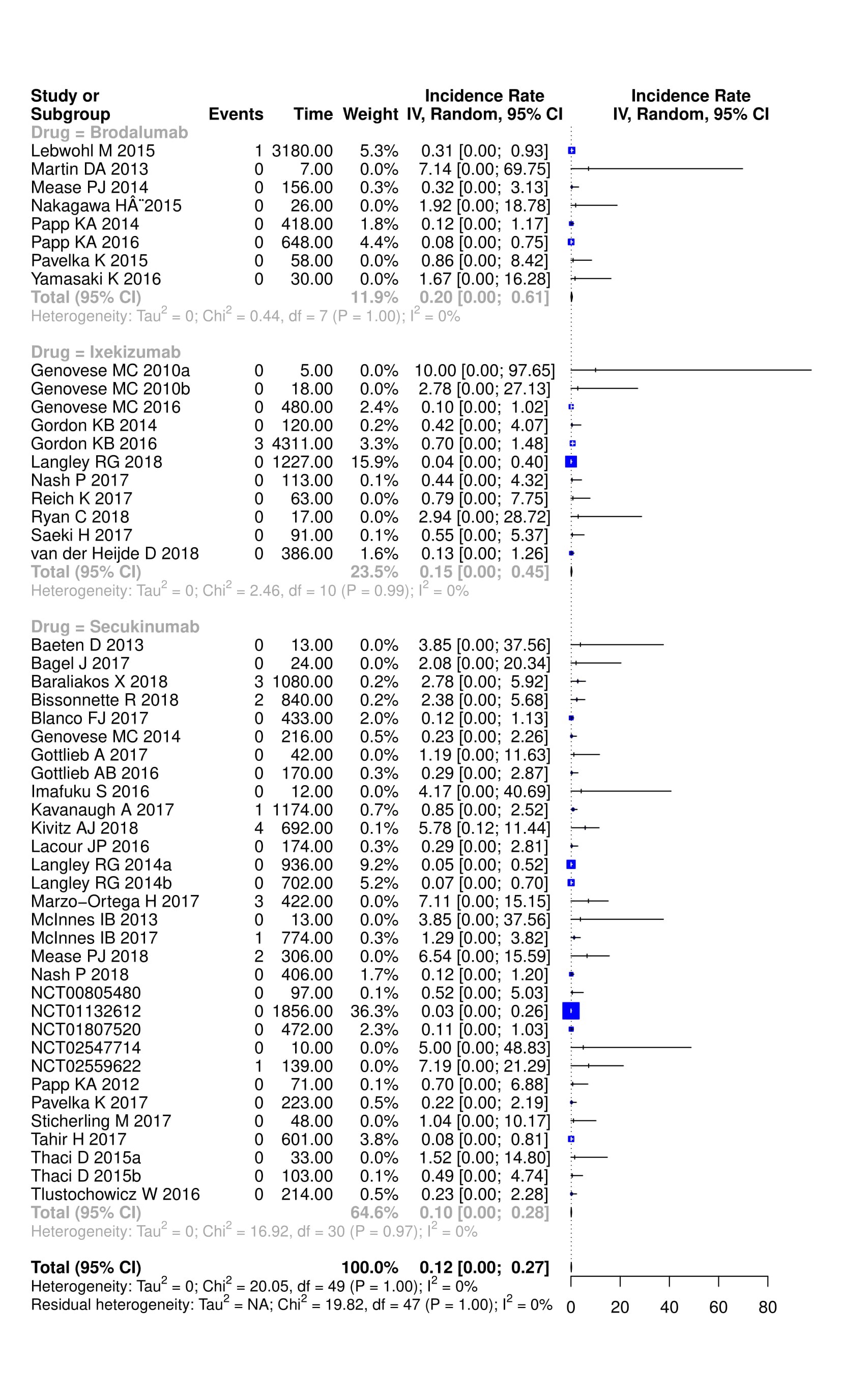

Supplement: S13 Fig — (JPG) [file pone.0233781.s014.jpg]

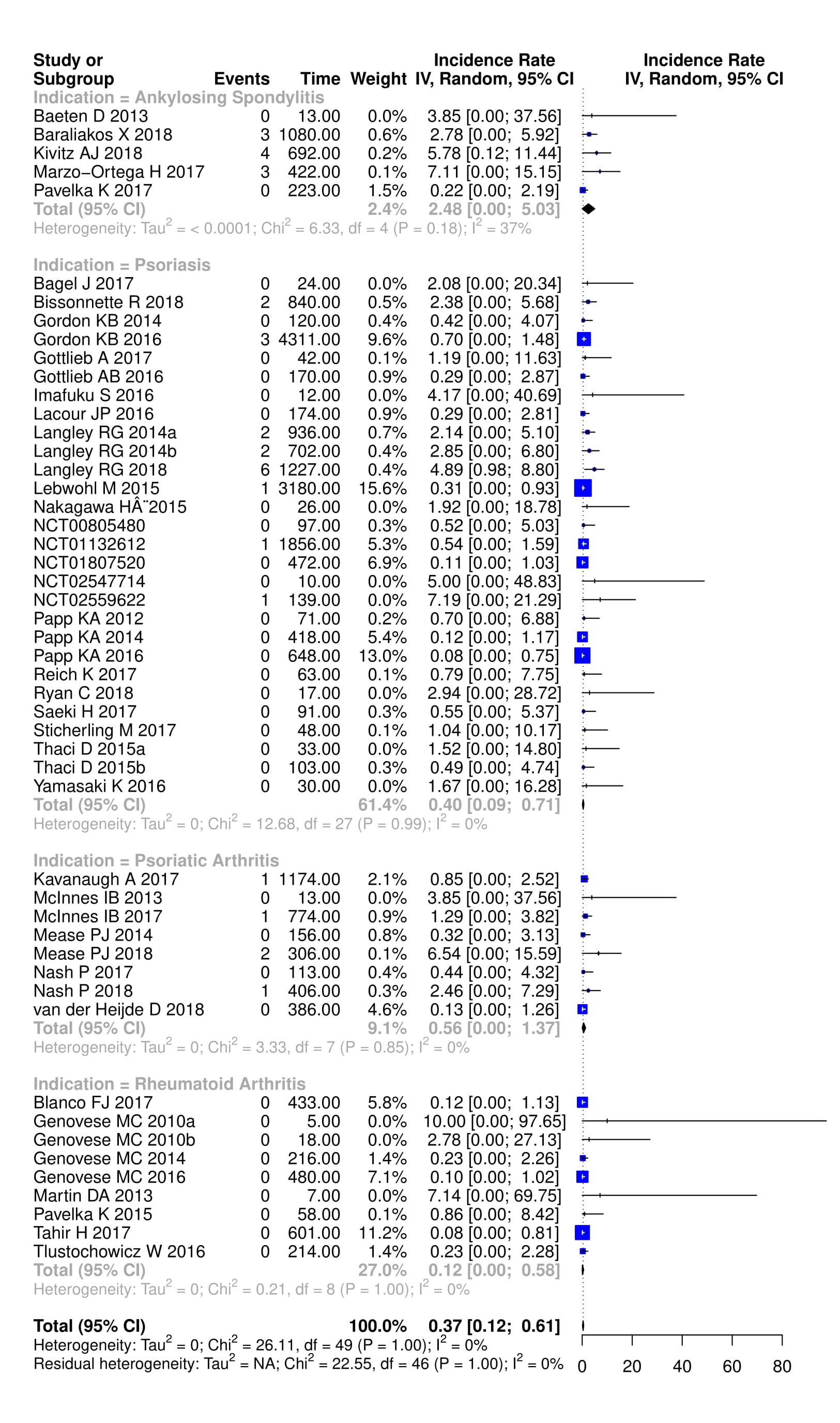

Supplement: S14 Fig — (JPG) [file pone.0233781.s015.jpg]

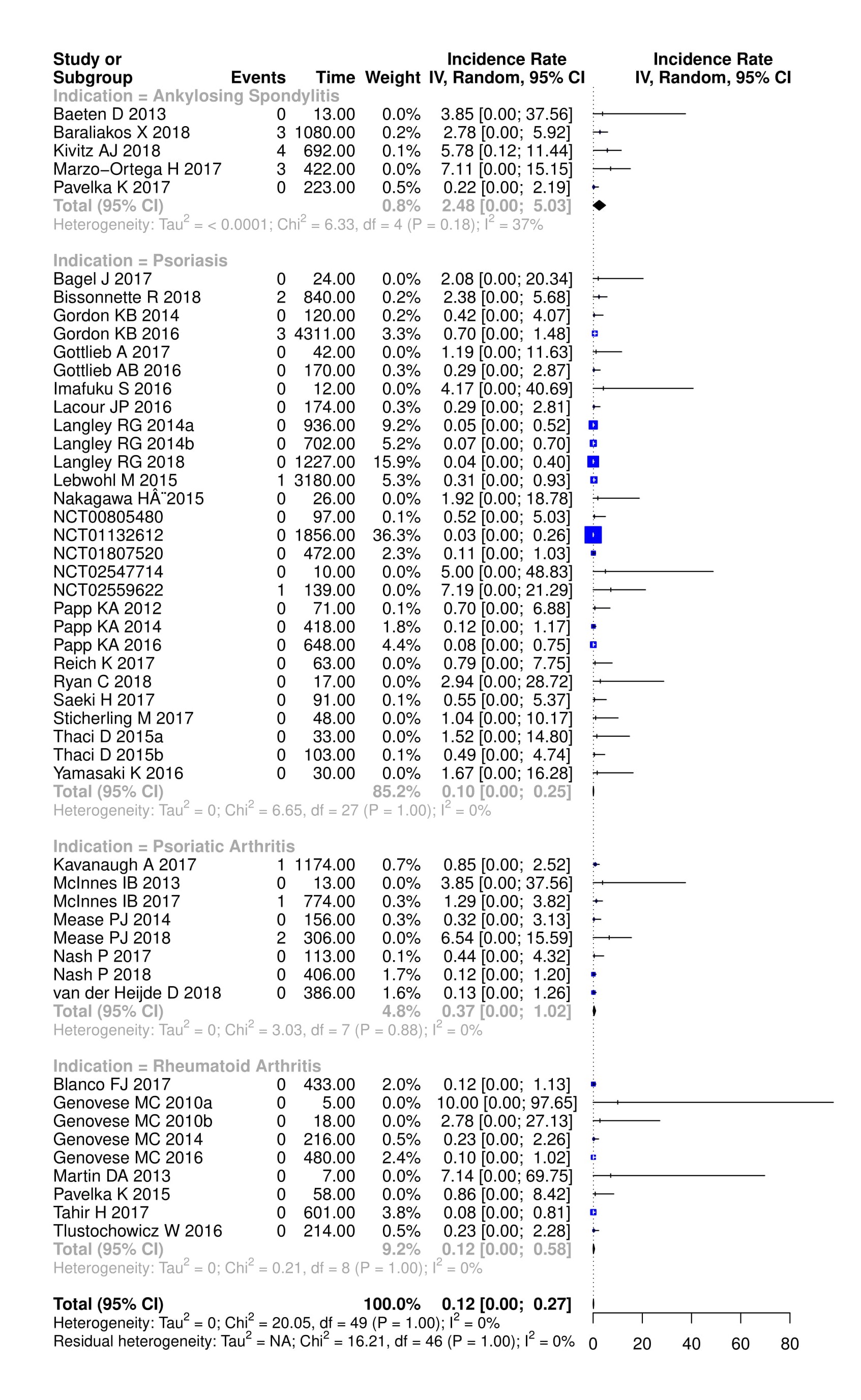

Supplement: S15 Fig — (JPG) [file pone.0233781.s016.jpg]

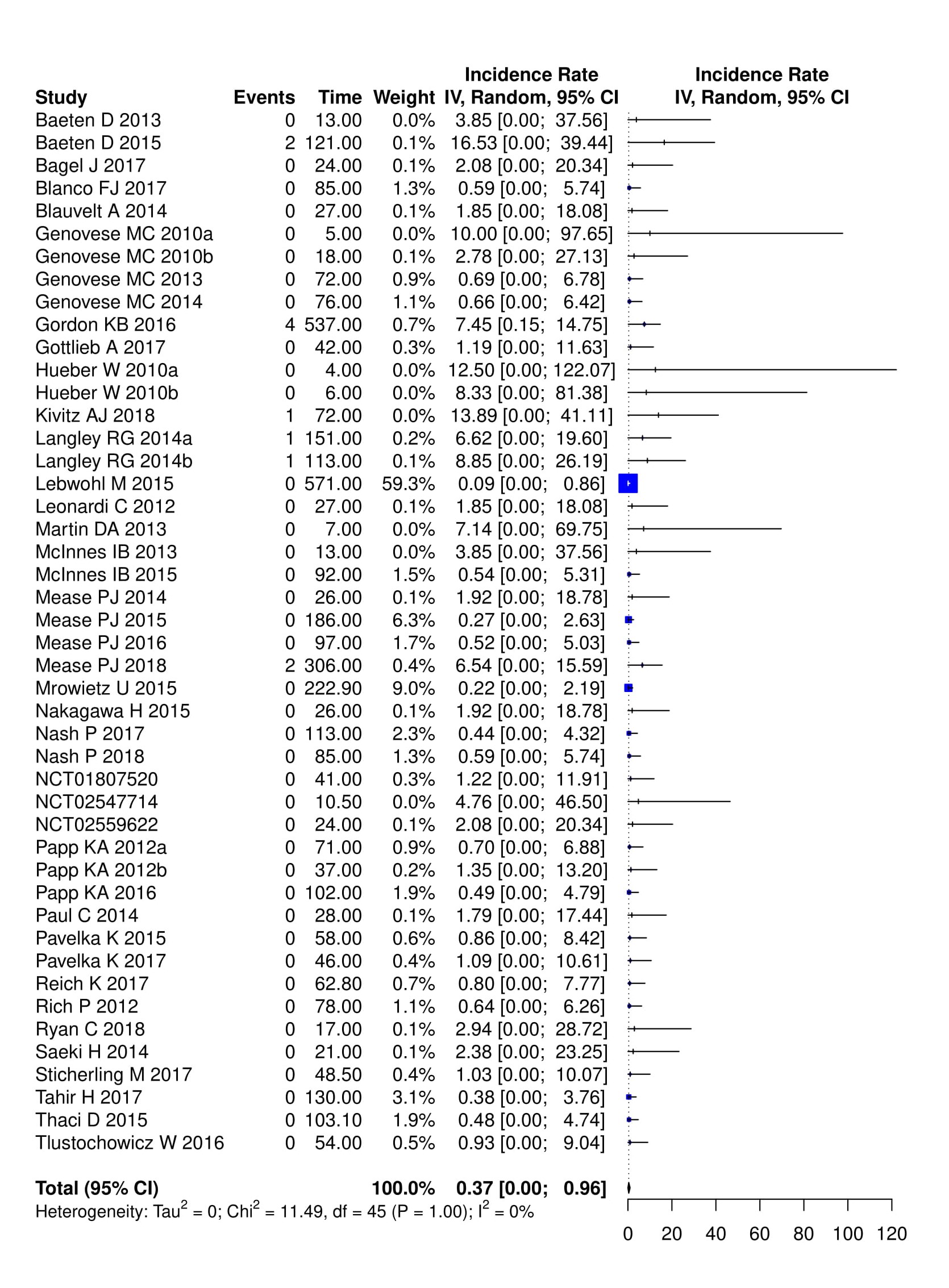

Supplement: S16 Fig — (JPG) [file pone.0233781.s017.jpg]

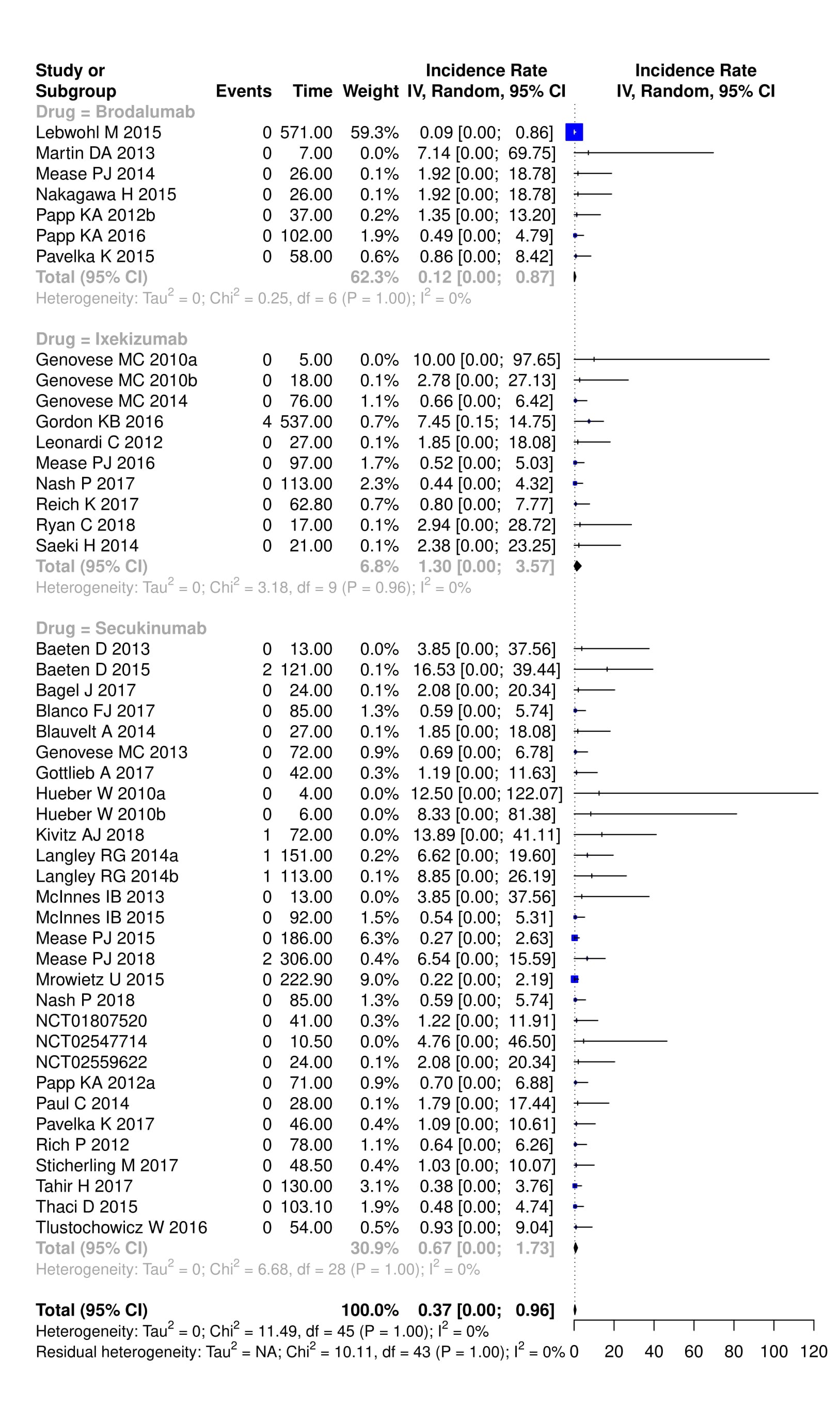

Supplement: S17 Fig — (JPG) [file pone.0233781.s018.jpg]

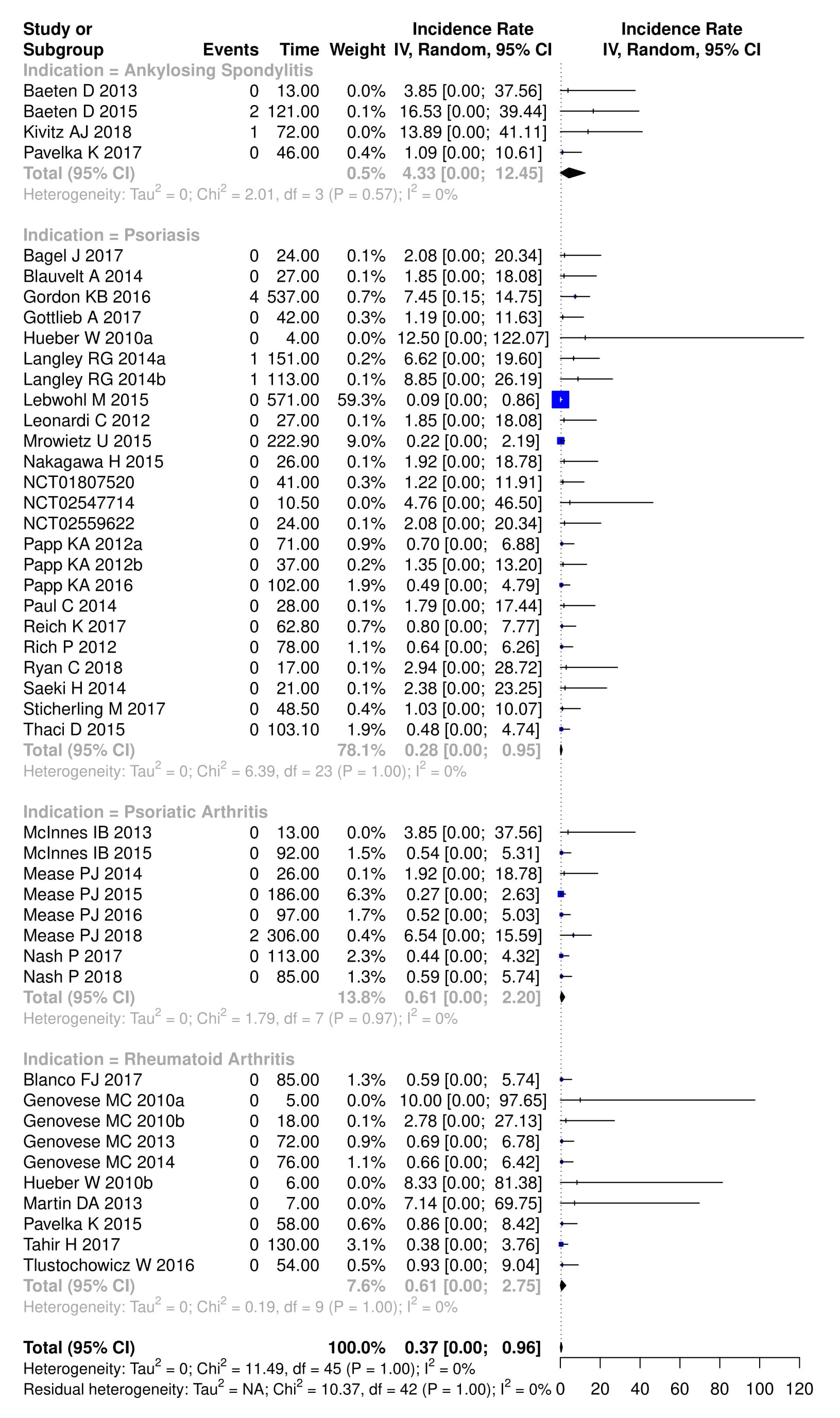

Supplement: S18 Fig — (JPG) [file pone.0233781.s019.jpg]
