# Supplementary material for: Risk for development of inflammatory bowel disease under inhibition of interleukin 17: A systematic review and meta-analysis
Source: PLoS One. 2020 May 27;15(5):e0233781. doi: 10.1371/journal.pone.0233781 (PMC7252630; doi:10.1371/journal.pone.0233781)
Supplement: S1 Table — (DOCX) [file pone.0233781.s020.docx]

| **Study title** | **NCT** | **Author** | **Year** | **Indication** | **Drug** | **description** | **treatment** |
| --- | --- | --- | --- | --- | --- | --- | --- |
|  | NCT00809614 | McInnes IB[[38](#_ENREF_38)] | 2013 | Psoriatic Arthritis | Secukinumab | Efficacy and safety of SEC | Randomization 2:1 to SEC 10mg/kg i.v. or placebo (two doses given 21 days apart) |
| FUTURE 1 | NCT01392326 | Mease PJ[[39](#_ENREF_39)] | 2015 | Psoriatic Arthritis | Secukinumab | Efficacy and safety of SEC | Randomization 1:1:1 to SEC 10mg/kg i.v. at weeks 0,2,4; afterwards SEC 150mg s.c. q4w versus SEC 75mg q4w versus placebo q4w; Rerandomization for patients on placebo to SEC 150mg s.c. q4w or SEC 75mg s.c. q4w at week 16 or week 24 |
| FUTURE 1 | NCT01392326 | Kavanaugh A[[40](#_ENREF_40)] | 2017 | Psoriatic Arthritis | Secukinumab | Efficacy and safety of SEC | Randomization 1:1:1 to SEC 10mg/kg i.v. at weeks 0,2,4; afterwards SEC 150mg s.c. q4w versus SEC 75mg q4w versus placebo q4w; Rerandomization for patients on placebo to SEC 150mg s.c. q4w or SEC 75mg s.c. q4w at week 16 or week 24 |
| FUTURE 2 | NCT01752634 | McInnes IB[[11](#_ENREF_11)] | 2015 | Psoriatic Arthritis | Secukinumab | Efficacy and safety of SEC | Randomization 1:1:1:1 to SEC 300mg s.c., 150mg s.c., 75mg s.c. or placebo s.c. weekly (week 0-4) and q4w after week 4 |
| FUTURE 2 | NCT01752634 | McInnes IB[[41](#_ENREF_41)] | 2017 | Psoriatic Arthritis | Secukinumab | Efficacy and safety of SEC | Randomization 1:1:1:1 to SEC 300mg s.c., 150mg s.c., 75mg s.c. or placebo s.c. weekly (week 0-4) and q4w after week 4; Rerandomization in the placebo group at week 16 or week 24 to SEC 300mg s.c. or SEC 150mg s.c. |
| FUTURE 3 | NCT01989468 | Nash P[[42](#_ENREF_42)] | 2018 | Psoriatic Arthritis | Secukinumab | Efficacy and safety of SEC administration by autoinjector | Randomization 1:1:1 to SEC 300mg s.c. or 150mg s.c. or placebo at week 0,1,2,3,4 and q4w after week 4; Rerandomization of patients on placebo to SEC 300mg s.c. or 150mg s.c. at week 16 or week 24 |
| FUTURE 5 | NCT02404350 | Mease PJ[[43](#_ENREF_43)] | 2018 | Psoriatic Arthritis | Secukinumab | Efficacy of SEC on clinical signs, symptoms and radiographic progression | Randomization 2:2:2:3 to SEC 300mg s.c. or 150mg s.c. with loading dose, SEC 150mg s.c. without loading dose or placebo |
| FIXTURE | NCT01358578 | Langley RG[[9](#_ENREF_9)] | 2014 | Plaque Psoriasis | Secukinumab | Efficacy and safety of SEC versus Placebo and versus Etanercept | SEC 300mg s.c. versus SEC 150mg s.c. versus Placebo versus Etanercept |
| ERASURE | NCT01365455 | Langley RG[[9](#_ENREF_9)] | 2014 | Plaque Psoriasis | Secukinumab | Efficacy and safety of SEC versus Placebo | SEC 300mg s.c. versus SEC 150mg s.c. versus Placebo |
| FEATURE | NCT01555125 | Blauvelt A[[44](#_ENREF_44)] | 2014 | Psoriasis | Secukinumab | Efficacy and safety of SEC versus Placebo | SEC 300mg s.c. versus SEC 150mg s.c. versus Placebo |
| FEATURE Maintainance | NCT01555125 | Gottlieb AB[[45](#_ENREF_45)] | 2016 | Psoriasis | Secukinumab | Efficacy and safety of SEC | SEC 300mg s.c. versus SEC 150mg s.c. versus Placebo |
| JUNCTURE | NCT01636687 | Paul C[[46](#_ENREF_46)] | 2015 | Psoriasis | Secukinumab | Efficacy and safety of SEC versus Placebo | SEC 300mg s.c. versus SEC 150mg s.c. versus Placebo |
| JUNCTURE Maintainance | NCT01636687 | Lacour JP[[47](#_ENREF_47)] | 2016 | Psoriasis | Secukinumab | Efficacy and safety of SEC versus Placebo | SEC 300mg s.c. versus SEC 150mg s.c. versus Placebo |
| SCULPTURE | NCT01406938 | Mrowietz U[[33](#_ENREF_33)] | 2015 | Psoriasis | Secukinumab | SEC retreatment-as needed versus fixed-interval maintenance regimen | SEC 300mg or 150mg at weeks 0,1,2,3,4,8; at weeks 12 rerandomization in PASI75 responders to: fixed interval or retreatment as needed (relapse); maintenance treatment in responders (PASI75): SEC 300mg retreatment-as-needed (n=217), SEC 300mg q4w fixed-interval (n=217), SEC 150mg retreatment-as-needed (n=206), SEC 150mg q4w fixed-interval (n=203) |
| SCULTPURE LTE | NCT01640951 | Bissonnette R[[34](#_ENREF_34)] | 2018 | Psoriasis | Secukinumab | Efficacy and safety of SEC | SEC 300mg s.c. q4w |
| STATURE | NCT01412944 | Thaci D[[48](#_ENREF_48)] | 2015 | Psoriasis | Secukinumab | SEC treatment in partial responders from SCULPTURE study | SEC 10mg/kg i.v. at week 0,2,4 versus SEC 300mg s.c. at week 0,4; then SEC 300mg s.c. q4w |
| A2211 | NCT00941031 | Rich P[[49](#_ENREF_49)] | 2012 | Plaque Psoriasis | Secukinumab | SEC dose and regimen finding study | Randomization 1:2:2:1 to SEC 150mg s.c. at week 0 versus week 0,4,8 versus week 0,1,2,4,8 versus matching placebo; Rerandomization in maintenance period to: fixed-interval, start-of-relapse regimen or open label |
| A2211E1 | NCT01132612 | Unpublished data* |  | Psoriasis | Secukinumab | Extension of A2211 | Maintainance treatment; 1) fixed interval regimen (150mg q12w) 2) treatment at start of relapse regimen (Secukinumab at start of relapse) 3) SEC q4w open label |
| A2220 | NCT01071252 | Papp KA[[50](#_ENREF_50)] | 2012 | Plaque Psoriasis | Secukinumab | SEC dose-ranging study | Randomization to Placebo, SEC 1x25mg s.c., 3x25mg s.c., 3x75mg s.c., 3x150mg s.c. or placebo at weeks 0,4,8 |
| A2212 | NCT00805480 | Unpublished data* |  | Psoriasis | Secukinumab | Multiple-loading dose regimen | SEC 3mg/kg on day 1 or SEC 10mg/kg on day 1 or SEC 10mg/kg on days 1,15,29 or placebo |
|  |  | Hueber W[[51](#_ENREF_51)] | 2010 | Psoriasis, Rheumatoid Arthritis, Uveitis | Secukinumab | Efficacy and safety of SEC | SEC weight based dosing versus placebo |
| TRANSFIGURE | NCT01807520 | Unpublished data* |  | Nail psoriasis | Secukinumab | Safety, Tolerability, and Efficacy of Secukinumab | In treatment period 1, participants were randomized in a 1:1:1 ratio to SEC 150mg, SEC 300mg or placebo; In treatment period 2, placebo participants were re-randomized in a 1:1 ratio to SEC 150mg or SEC 300mg |
| CARIMA | NCT02559622 | Unpublished data* |  | Psoriasis | Secukinumab | effect of SEC on endothelial dysfunction and arterial stiffness | SEC 300mg s.c. q4w versus SEC 150mg q4w versus PCB until week 12 followed by SEC 300mg s.c. q4w versus PCB until week 12 followed by SEC 150mg s.c. |
| PRIME | NCT02474082 | Sticherling M[[52](#_ENREF_52)] | 2017 | Psoriasis | Secukinumab | SEC versus FAE in plaquepsoriasis | SEC 300mg s.c. versus oral FAE |
| JP01 | NCT02547714 | Unpublished data* |  | Psoriasis | Secukinumab | Efficacy and safety of SEC | SEC 300mg s.c. on weeks 0,1,2,3,4,8,12, open label |
| SCALP | NCT02267135 | Bagel J[[53](#_ENREF_53)] | 2017 | Scalppsoriasis | Secukinumab | Efficacy and safety of SEC | SEC 300mg versus PCB, PCB non-responders switched to SEC 300mg |
| CLEAR | NCT02074982 | Thaci D[[54](#_ENREF_54)] | 2015 | Psoriasis | Secukinumab | Efficacy and safety | SEC 300mg or ustekinumab per label |
| GESTURE | NCT01806597 | Gottlieb A[[55](#_ENREF_55)] | 2017 | Palmoplantar Psoriasis | Secukinumab | Efficacy and safety of SEC | Randomization 1:1:1 To SEC 300mg, 150mg or PCB |
|  | NCT01952015 | Imafuku S[[56](#_ENREF_56)] | 2016 | Pustular Psoriasis | Secukinumab | Efficacy and safety | SEC 150mg s.c. at weeks 0,1,2,3,4 and q4w thereafter |
|  | NCT00809159 | Baeten D[[57](#_ENREF_57)] | 2013 | Ankylosing Spondylitis | Secukinumab | Proof of concept, efficacy and safety of SEC | Randomization 4:1 to SEC 2x10mg/kg i.v. versus PCB |
| MEASURE 1+2 | NCT01358175 and NCT01649375 | Baeten D[[12](#_ENREF_12)] | 2015 | Ankylosing Spondylitis | Secukinumab | Efficacy and safety of SEC | MEASURE 1: Randomization to SEC 10mg/kg i.v. or PCB at week 0,2,4 followed by SEC 150mg s.c. or 75mg s.c. or PCB q4w; MEASURE 2: SEC 150mg s.c. or 75mg s.c. or PCB at week 0,1,2,3 and every 4 weeks starting week 4; Rerandomization at week 16 in both studies of patients under PCB to SEC 150mg s.c. or 75mg s.c. |
| MEASURE 1 | NCT01863732 | Baraliakos X[[58](#_ENREF_58)] | 2018 | Ankylosing Spondylitis | Secukinumab | Efficacy and safety of SEC | MEASURE 1: Randomization to SEC 10mg/kg i.v. or PCB at week 0,2,4 followed by SEC 150mg s.c. or 75mg s.c. or PCB q4w; Rerandomization at week 16 of patients under PCB to SEC 150mg s.c. or 75mg s.c. |
| MEASURE 1 | NCT01358175 | Braun J[[59](#_ENREF_59)] | 2016 | Ankylosing Spondylitis | Secukinumab | Efficacy and safety of SEC | MEASURE 1: Randomization to SEC 10mg/kg i.v. or placebo at week 0,2,4 followed by SEC 150mg s.c. or 75mg s.c. or placebo q4w; Rerandomization at week 16 of patients under placebo to SEC 150mg s.c. or 75mg s.c. |
| MEASURE 2 | NCT01649375 | Marzo-Ortega H[[60](#_ENREF_60)] | 2017 | Ankylosing Spondylitis | Secukinumab | Efficacy and safety of SEC | SEC 150mg s.c. or 75mg s.c. or placebo at week 0,1,2,3 and every 4 weeks starting week 4; Rerandomization at week 16 of patients under placebo to SEC 150mg s.c. or 75mg s.c. |
| MEASURE 2 | NCT01649375 | Marzo-Ortega H[[32](#_ENREF_32)] | 2017 | Ankylosing Spondylitis | Secukinumab | Efficacy and safety of SEC | SEC 150mg s.c. or 75mg s.c. or placebo at week 0,1,2,3 and every 4 weeks starting week 4; Rerandomization at week 16 of patients under placebo to SEC 150mg s.c. or 75mg s.c. |
| MEASURE 3 | NCT02008916 | Pavelka K[[61](#_ENREF_61)] | 2017 | Ankylosing Spondylitis | Secukinumab | Efficacy and safety of SEC | Randomization to SEC 10mg/kg i.v. or placebo at week 0,2,4 followed by SEC 150mg s.c. or 75mg s.c. or placebo q4w; Rerandomization at week 16 of patients under placebo to SEC 150mg s.c. or 75mg s.c. |
| MEASURE 4 | NCT02159053 | Kivitz AJ[[62](#_ENREF_62)] | 2018 | Ankylosing Spondylitis | Secukinumab | Efficacyandsafety | Randomization 1:1:1 to SEC 150mg s.c. with loading dose, without loading dose or placebo; at week 16 patients on placebo were switched to SEC 150mg s.c. q4w |
|  | NCT00928512 | Genovese MC[[63](#_ENREF_63)] | 2013 | Rheumatoid Arthritis | Secukinumab | Efficacy and safety of SEC | Randomization to SEC 25mg s.c., 75mg s.c., 150mg s.c., 300mg s.c. or placebo |
|  | NCT00928512 | Genovese MC[[64](#_ENREF_64)] | 2014 | Rheumatoid Arthritis | Secukinumab | Dose findingstudy | Randomization to SEC 25mg s.c., 75mg s.c., 150mg s.c., 300mg s.c. or placebo; Treatment escalation in non-responders at week 20 |
|  | NCT01359943 | Tlustochowicz W[[65](#_ENREF_65)] | 2016 | Rheumatoid Arthritis | Secukinumab | Efficacy and safety of SEC | Randomization 2:2:1 to SEC 10mg/kg i.v. at weeks 0,2,4 and 150mg s.c. q4w thereafter or SEC 150mg s.c. once weekly for 5 weeks and 150mg s.c. thereafter or placebo; patients under placebo were switched to SEC 150mg s.c. q4w at week 16 |
|  | NCT01350804 | Blanco FJ[[66](#_ENREF_66)] | 2017 | Rheumatoid Arthritis | Secukinumab | Efficacy and safety of SEC | Randomization 1:1:1:1 to SEC 10mg/kg i.v. at weeks 0,2,4 and either SEC 150mg s.c. or 75mg s.c. q4w thereafter or abatacept or placebo; Rerandomization to SEC 150mg s.c. or 75mg s.c. in placebo and abatacept group depending on response (week 16 or 24) |
| REASSURE | NCT01377012 | Tahir H[[67](#_ENREF_67)] | 2017 | Rheumatoid Arthritis | Secukinumab | Efficacy and safety of SEC | Randomization to SEC 10mg/kg i.v. at weeks 0,2,4 and SEC 150mg s.c. or 75mg s.c. q4w or matching placebo; in placebo arm escape treatment or rerandomization at week 24 to SEC 150mg s.c. or 75mg |
|  | NCT01107457 | Leonardi C[[68](#_ENREF_68)] | 2012 | Psoriasis | Ixekizumab | Dose finding study of IXE | Randomization to IXE 10,25,75,150mg or placebo at weeks 0,2,4,8,12,16 |
|  | NCT01107457 | Gordon KB[[69](#_ENREF_69)] | 2014 | Psoriasis | Ixekizumab | Efficacy and safety of IXE | After induction study; patients with PASI75 entered treatment free period until week32 and patients without PASI75 entered OLE: IXE 120mg s.c. q4w |
| UNCOVER 1+2+3 | NCT0147451, NCT01597245, NCT01646177 | Gordon KB[[35](#_ENREF_35)] | 2016 | Psoriasis | Ixekizumab | Efficacy and safety of IXE | Randomization to IXE 160mg s.c. starting dose and IXE 80mg s.c. q2w or IXE 160mg s.c. starting dose and IXE 80mg s.c. q4w or matching placebo. Additional cohorts in UNCOVER-2/3 received 50mg of etanercept twice weekly; At week 12: in UNCOVER-1/2 responders were rerandomized to IXE 80mg s.c. q4w, 80mg s.c. q2w or placebo. In UNCOVER-3 patients entered LTE with IXE 80mg s.c. q4w trough week 60 |
| UNCOVER 3 | NCT01646177 | Blauvelt A[[36](#_ENREF_36)] | 2017 | Psoriasis | Ixekizumab | Efficacy and safety | |
| UNCOVER 3 | NCT01646177 | Leonardi C[[37](#_ENREF_37)] | 2018 | Psoriasis | Ixekizumab | Efficacy and safety | IXE 80mg s.c. q4w, IXE 80mg s.c. q2w possible after week 60 |
| UNCOVER-J | NCT01624233 | Saeki H[[70](#_ENREF_70)] | 2015 | Plaque Psoriasis, erythrodermic psoriasis, generalized pustular psoriasis | Ixekizumab | Efficacy and safety of open label IXE | IXE 160mg s.c. at week 0, 80mg s.c. q2w until week 12 and 80mg s.c. q4w until week 24 |
| UNCOVER-J | NCT01624233 | Saeki H[[71](#_ENREF_71)] | 2017 | Psoriasis | Ixekizumab | Efficacy and safety of IXE | IXE 80mg s.c. q4w |
| IXORA-P | NCT02513550 | Langley RG[[72](#_ENREF_72)] | 2018 | Plaque Psoriasis | Ixekizumab | Efficacy and safety of IXE under continuous q2w dosing | Randomization 2:1:1 to IXE 80mg s.c. q2w, q4w or dose adjustement per protocol after initial loading dose (IXE 160mg s.c. at baseline) |
| IXORA-Q | NCT02718898 | Ryan C[[73](#_ENREF_73)] | 2018 | Genital Psoriasis | Ixekizumab | Efficacy and safety | Randomization 1:1 to IXE 160mg at week 0 and 80mg q2w thereafter or placebo |
| IXORA-S | NCT02561806 | Reich K[[74](#_ENREF_74)] | 2017 | Psoriasis | Ixekizumab | Efficacy and safety | IXE 160mg starting dose, 80mg q2w until week 12 and q4w thereafter versus ustekinumab |
| SPIRIT-P1 | NCT01695239 | Mease PJ[[75](#_ENREF_75)] | 2017 | Psoriatic Arthritis | Ixekizumab | Safety and Efficacy | Randomization 1:1:1:1 to IXE 160mg s.c. at baseline and IXE 80mg s.c. q4w thereafter, 80mg s.c. q2w thereafter, adalimumab 40mg s.c. q4w or placebo |
| SPIRIT-P2 | NCT02349295 | Nash P[[76](#_ENREF_76)] | 2017 | Psoriatic Arthritis | Ixekizumab | Safety and Efficacy | Randomization 1:1:1 to IXE 160mg s.c. at baseline and IXE 80mg s.c. q4w or q2w thereafter of matching placebo |
| SPIRIT-P1 | NCT01695239 | van der Heijde D[[77](#_ENREF_77)] | 2018 | Psoriatic Arthritis | Ixekizumab | Safety and Efficacy | At week 16/week 24 patients on adalimumab or placebo were rerandomized 1:1 to IXE 80mg s.c. q2w or IXE 80mg s.c. q4w |
|  | N/A | Genovese MC[[78](#_ENREF_78)] | 2010 | Rheumatoid Arthritis | Ixekizumab | Safety, tolerability, pharmacokinetics, pharmacodynamics and efficacy | Part A: Randomization to one i.v. dose: 0.06, 0.2, 0.6 or 2.0 mg/kg or placebo; Part B: Randomization to one i.v. dose (0.2, 0.6, 2.0 mg/kg) or placebo q2w for five total doses |
|  | NCT00966875 | Genovese MC[[79](#_ENREF_79)] | 2014 | Rheumatoid Arthritis | Ixekizumab | Safety and efficacy, Dose finding study | bDMARD-naiv: Randomization 3:2:2:2:3:2 to Placebo or IXE 3mg,10mg,30mg,80mg,180mg s.c. Prior aTNFα use: Randomization 1:1:1 to Placebo or IXE 80mg or 180mg s.c. at weeks 0,1,2,4,6,8,10 |
|  | NCT00966875 | Genovese MC[[80](#_ENREF_80)] | 2016 | Rheumatoid Arthritis | Ixekizumab | Safety and efficacy | IXE 160mg s.c. at weeks 16,18,20 and q4w thereafter |
|  | NCT00975637 | Papp KA[[81](#_ENREF_81)] | 2012 | Psoriasis | Brodalumab | Safety and efficacy, Dose finding study | BRO 70mg, 140mg, 210 mg or placebo s.c. at weeks 0,1,2,4,6,8,10 or BRO 280mg s.c. at weeks 0,4,8 |
|  | NCT01101100 | Papp KA[[82](#_ENREF_82)] | 2014 | Psoriasis | Brodalumab | Safety and efficacy | BRO 210mg q2w with protocol amendment (140mg q2w for patients with < 100kg body weight) |
|  | NCT01748539 | Nakagawa H[[83](#_ENREF_83)] | 2015 | Plaque Psoriasis incl. Psoriatic arthritis | Brodalumab | Safety and efficacy | BRO 70mg, 140mg, 210 mg or placebo s.c. at weeks 0,1,2,4,6,8,10 |
| AMAGINE-1 | NCT01708590 | Papp KA[[84](#_ENREF_84)] | 2016 | Psoriasis | Brodalumab | Safety and efficacy | Randomization to BRO 140mg, 210 mg or placebo s.c.q2w with an additional dose at week 1; Rerandomization at week 12 to BRO 140mg, 210mg or placebo s.c. q2w per protocol |
| AMAGINE-2+3 | NCT01708603, NCT01708629 | Lebwohl M[[13](#_ENREF_13)] | 2015 | Psoriasis | Brodalumab | Safety and efficacy | Randomization 2:2:1:1 to BRO 210mg or 140mg s.c. at weeks 0,1,2,4,6,8,10 or ustekinumab or placebo; Rerandomization 2:2:2:1 of patients on BRO at week 12 to BRO 210mg q2w s.c. or 140mg q2w s.c. or 140mg q4w s.c. or 140mg q8w s.c.; patients on placebo received BRO 210mg q2w |
|  | NCT01782937 | Yamasaki K[[85](#_ENREF_85)] | 2016 | Generalized pustular psoriasis and psoriatic erythroderma | Brodalumab | Efficacy and Safety | open label BRO 140mg s.c. at weeks 0,1,2 and q2w thereafter |
|  | NCT01516957 | Mease PJ[[86](#_ENREF_86)] | 2014 | Psoriatic Arthritis | Brodalumab | Efficacy and safety | Randomization 1:1:1 to BRO 140mg, 280mg or placebo at weeks 0,1,2,4,6,8,10; open label BRO at week 12: BRO 280mg q2w |
|  | NCT00771030 | Martin DA[[87](#_ENREF_87)] | 2013 | Rheumatoid Arthritis | Brodalumab | Safety, pharmacokinetics and clinical response | Randomization 3:1 to ascending doses of BRO (50, 140, 210mg) or placebo or BRO i.v. (420mg or 700mg) |
|  | NCT00950989 | Pavelka K[[88](#_ENREF_88)] | 2015 | Rheumatoid Arthritis | Brodalumab | Safety, tolerability and efficacy | BRO 70mg, 140mg, 210mg s.c. or placebo |

* data received from the European Medical Agency

SEC – Secukinumab; q4w – every four weeks; PASI –Psoriasis Area and Severity Index; q12w – every twelve weeks; PCB – placebo; FAE – fumaricacid esters; IXE – ixekizumab; OLE – open label extension; q2w – every twelve weeks; LTE – long term extension; bDMARD – biologic disease modifying anti-rheumatic drug; aTNFα – anti-Tumor necrosis factorα; BRO – brodalu
