# Supplementary material for: Risk for development of inflammatory bowel disease under inhibition of interleukin 17: A systematic review and meta-analysis
Source: PLoS One. 2020 May 27;15(5):e0233781. doi: 10.1371/journal.pone.0233781 (PMC7252630; doi:10.1371/journal.pone.0233781)
Supplement: S2 Table — (DOCX) [file pone.0233781.s021.docx]

**Risk of bias assessment for randomized controlled trials**

Table 1.1: Risk of Bias Assessment for *McInnes IB, 2013 (NCT00809614)*

| Domain | Risk of Bias | Comments |
| --- | --- | --- |
| Sequence generation | Low | - Randomization done using validated system |
| Allocation concealment | Low | - Central allocation |
| Blinding of participants, personnel and outcome assessors | Low | - Patients and investigators were blinded to treatment allocation |
| Incomplete outcome data | Low | - All patients accounted for |
| Selective outcome reporting | Low | - All of study’s pre-specified outcomes have been reported |
| Other sources of bias | Low | - The study appears to be free of other sources of bias |

Table 1.2: Risk of Bias Assessment for *Mease PJ, 2015 (NCT01392326)*

| Domain | Risk of Bias | Comments |
| --- | --- | --- |
| Sequence generation | Low | - Randomization done using an interactive voice-Web response system |
| Allocation concealment | Low | - Central allocation |
| Blinding of participants, personnel and outcome assessors | Low | - Patients and investigators were blinded to treatment allocation |
| Incomplete outcome data | Low | - All patients accounted for |
| Selective outcome reporting | Low | - All of study’s pre-specified outcomes have been reported |
| Other sources of bias | Low | - The study appears to be free of other sources of bias |

Table 1.3: Risk of Bias Assessment for *Kavanaugh A, 2017 (NCT01752634)*

| Domain | Risk of Bias | Comments |
| --- | --- | --- |
| Sequence generation | Low | - Randomization performed according to system used in FUTURE I |
| Allocation concealment | Low | - Central allocation |
| Blinding of participants, personnel and outcome assessors | Low | - Patients and investigators were blinded to treatment allocation |
| Incomplete outcome data | Low | - All patients accounted for |
| Selective outcome reporting | Low | - All of study’s pre-specified outcomes have been reported |
| Other sources of bias | Low | - The study appears to be free of other sources of bias |

Table 1.4: Risk of Bias Assessment for *McInnes IB, 2015 (NCT01752634)*

| Domain | Risk of Bias | Comments |
| --- | --- | --- |
| Sequence generation | Low | - Randomization done using interactive voice or web response system |
| Allocation concealment | Low | - Central allocation |
| Blinding of participants, personnel and outcome assessors | Low | - Patients and investigators were blinded to treatment allocation |
| Incomplete outcome data | Low | - All patients accounted for |
| Selective outcome reporting | Low | - All of study’s pre-specified outcomes have been reported |
| Other sources of bias | Low | - The study appears to be free of other sources of bias |

Table 1.5: Risk of Bias Assessment for *McInnes IB, 2017 (NCT01752634)*

| Domain | Risk of Bias | Comments |
| --- | --- | --- |
| Sequence generation | Low | - Randomization performed according to system used in FUTURE 2 |
| Allocation concealment | Low | - Central allocation |
| Blinding of participants, personnel and outcome assessors | Low | - Patients and investigators were blinded to treatment allocation |
| Incomplete outcome data | Low | - All patients accounted for |
| Selective outcome reporting | Low | - All of study’s pre-specified outcomes have been reported |
| Other sources of bias | Low | - The study appears to be free of other sources of bias |

Table 1.6: Risk of Bias Assessment for *Nash P, 2018 (NCT01989468)*

| Domain | Risk of Bias | Comments |
| --- | --- | --- |
| Sequence generation | Low | - Randomization done using interactive response technology (IRT) |
| Allocation concealment | Low | - Central allocation |
| Blinding of participants, personnel and outcome assessors | Low | - Patients and investigators were blinded to treatment allocation |
| Incomplete outcome data | Low | - All patients accounted for |
| Selective outcome reporting | Low | - All of study’s pre-specified outcomes have been reported |
| Other sources of bias | Low | - The study appears to be free of other sources of bias |

Table 1.7: Risk of Bias Assessment for *Mease PJ, 2018 (NCT02404350)*

| Domain | Risk of Bias | Comments |
| --- | --- | --- |
| Sequence generation | Low | - Randomization done using interactive response technology (IRT) |
| Allocation concealment | Low | - Central allocation |
| Blinding of participants, personnel and outcome assessors | Low | - Patients and investigators were blinded to treatment allocation |
| Incomplete outcome data | Low | - All patients accounted for |
| Selective outcome reporting | Low | - All of study’s pre-specified outcomes have been reported |
| Other sources of bias | Low | - The study appears to be free of other sources of bias |

Table 1.8: Risk of Bias Assessment for *Langley RG, 2014 (NCT02404350 and NCT01365455)*

| Domain | Risk of Bias | Comments |
| --- | --- | --- |
| Sequence generation | Low | - Randomization done using validated system |
| Allocation concealment | Low | - Central allocation |
| Blinding of participants, personnel and outcome assessors | Low | - Patients and investigators were blinded to treatment allocation |
| Incomplete outcome data | Low | - All patients accounted for |
| Selective outcome reporting | Low | - All of study’s pre-specified outcomes have been reported |
| Other sources of bias | Low | - The study appears to be free of other sources of bias |

Table 1.9: Risk of Bias Assessment for *Blauvelt A, 2014 (NCT01555125)*

| Domain | Risk of Bias | Comments |
| --- | --- | --- |
| Sequence generation | Low | - Randomization done using interactive response technology (IRT) |
| Allocation concealment | Low | - Central allocation |
| Blinding of participants, personnel and outcome assessors | Low | - Patients and investigators were blinded to treatment allocation |
| Incomplete outcome data | Low | - All patients accounted for |
| Selective outcome reporting | Low | - All of study’s pre-specified outcomes have been reported |
| Other sources of bias | Low | - The study appears to be free of other sources of bias |

Table 1.10: Risk of Bias Assessment for *Gottlieb AB, 2016 (NCT01555125)*

| Domain | Risk of Bias | Comments |
| --- | --- | --- |
| Sequence generation | Low | - Randomization and rerandomization done using validated system |
| Allocation concealment | Low | - Central allocation |
| Blinding of participants, personnel and outcome assessors | Low | - Patients and investigators were blinded to treatment allocation |
| Incomplete outcome data | Low | - All patients accounted for |
| Selective outcome reporting | Low | - All of study’s pre-specified outcomes have been reported |
| Other sources of bias | Low | - The study appears to be free of other sources of bias |

Table 1.11: Risk of Bias Assessment for *Paul C, 2015 (NCT01636687)*

| Domain | Risk of Bias | Comments |
| --- | --- | --- |
| Sequence generation | Low | - Randomization done using validated system |
| Allocation concealment | Low | - Central allocation |
| Blinding of participants, personnel and outcome assessors | Low | - Patients and investigators were blinded to treatment allocation |
| Incomplete outcome data | Low | - All patients accounted for |
| Selective outcome reporting | Low | - All of study’s pre-specified outcomes have been reported |
| Other sources of bias | Low | - The study appears to be free of other sources of bias |

Table 1.12: Risk of Bias Assessment for *Lacour JP, 2016 (NCT01636687)*

| Domain | Risk of Bias | Comments |
| --- | --- | --- |
| Sequence generation | Low | - Rerandomization done using validated system |
| Allocation concealment | Low | - Central allocation |
| Blinding of participants, personnel and outcome assessors | Low | - Patients and investigators were blinded to treatment allocation |
| Incomplete outcome data | Low | - All patients accounted for |
| Selective outcome reporting | Low | - All of study’s pre-specified outcomes have been reported |
| Other sources of bias | Low | - The study appears to be free of other sources of bias |

Table 1.13: Risk of Bias Assessment for *Mrowietz U, 2015 (NCT01406938)*

| Domain | Risk of Bias | Comments |
| --- | --- | --- |
| Sequence generation | Low | - Rerandomization done using validated system |
| Allocation concealment | Low | - Central allocation |
| Blinding of participants, personnel and outcome assessors | Low | - Patients and investigators were blinded to treatment allocation |
| Incomplete outcome data | Low | - All patients accounted for |
| Selective outcome reporting | Low | - All of study’s pre-specified outcomes have been reported |
| Other sources of bias | Low | - The study appears to be free of other sources of bias |

Table 1.14: Risk of Bias Assessment for *Bissonnette R, 2018 (NCT01640951)*

| Domain | Risk of Bias | Comments |
| --- | --- | --- |
| Sequence generation | Low | - Initial randomization done using validated system |
| Allocation concealment | Low | - Central allocation |
| Blinding of participants, personnel and outcome assessors | Low | - Patients and investigators were blinded to treatment allocation in the beginning of the study up to the end of Year 3, open-label from Year 4 |
| Incomplete outcome data | Low | - All patients accounted for |
| Selective outcome reporting | Low | - All of study’s pre-specified outcomes have been reported |
| Other sources of bias | Low | - The study appears to be free of other sources of bias |

Table 1.15: Risk of Bias Assessment for *Thaci D, 2015 (NCT01412944)*

| Domain | Risk of Bias | Comments |
| --- | --- | --- |
| Sequence generation | Low | - Randomization done using interactive response technology (IRT) |
| Allocation concealment | Low | - Central allocation |
| Blinding of participants, personnel and outcome assessors | Low | - Patients and investigators were blinded to treatment allocation |
| Incomplete outcome data | Low | - All patients accounted for |
| Selective outcome reporting | Low | - All of study’s pre-specified outcomes have been reported |
| Other sources of bias | Low | - The study appears to be free of other sources of bias |

Table 1.16: Risk of Bias Assessment for *Rich P, 2012 (NCT00941031)*

| Domain | Risk of Bias | Comments |
| --- | --- | --- |
| Sequence generation | Low | - Randomization done using interactive response technology (IRT) |
| Allocation concealment | Low | - Central allocation |
| Blinding of participants, personnel and outcome assessors | Low | - Patients and investigators were blinded to treatment allocation, after the primary outcome analysis, the study sponsor team was unblended, the patients, local monitors, investigator staff and persons performing the assessments remained blinded |
| Incomplete outcome data | Low | - All patients accounted for |
| Selective outcome reporting | Low | - All of study’s pre-specified outcomes have been reported |
| Other sources of bias | Low | - The study appears to be free of other sources of bias |

Table 1.17: Risk of Bias Assessment for *study A2211E1 (NCT01132612)*

| Domain | Risk of Bias | Comments |
| --- | --- | --- |
| Sequence generation | Low | - Randomization done using interactive response technology (IRT) in initial induction trials, treatment was continued in maintenance trial according to initial randomization |
| Allocation concealment | Unclear | - No information |
| Blinding of participants, personnel and outcome assessors | Low | - Patients and investigators were blinded to treatment allocation, after the primary outcome analysis, the study sponsor team was unblended, the patients, local monitors, investigator staff and persons performing the assessments remained blinded |
| Incomplete outcome data | Low | - All patients accounted for |
| Selective outcome reporting | Unclear | - Adverse results have been reported online on clinicaltrials.gov, however statistical analysis was not conducted and published |
| Other sources of bias | High | - The trial did not get published in a peer-reviewed journal |

Table 1.18: Risk of Bias Assessment for *Papp KA, 2012 (NCT01071252)*

| Domain | Risk of Bias | Comments |
| --- | --- | --- |
| Sequence generation | Low | - Randomization done using interactive response technology (IRT) |
| Allocation concealment | Low | - Central allocation |
| Blinding of participants, personnel and outcome assessors | Low | - Patients and investigators were blinded to treatment allocation, after the primary outcome analysis, the study sponsor team was unblended, the patients, local monitors, investigator staff and persons performing the assessments remained blinded |
| Incomplete outcome data | Low | - All patients accounted for |
| Selective outcome reporting | Low | - All of study’s pre-specified outcomes have been reported |
| Other sources of bias | Low | - The study appears to be free of other sources of bias |

Table 1.19: Risk of Bias Assessment for *study A2212 (NCT00805480)*

| Domain | Risk of Bias | Comments |
| --- | --- | --- |
| Sequence generation | Unclear | - No information provided |
| Allocation concealment | Unclear | - No information provided |
| Blinding of participants, personnel and outcome assessors | Low | - Double blinded study |
| Incomplete outcome data | Low | - All patients accounted for |
| Selective outcome reporting | Unclear | - Adverse results have been reported online on clinicaltrials.gov, however statistical analysis was not conducted and published |
| Other sources of bias | Low | - The study appears to be free of other sources of bias |

Table 1.20: Risk of Bias Assessment for *Hueber W, 2010*

| Domain | Risk of Bias | Comments |
| --- | --- | --- |
| Sequence generation | High | - No clear statement on randomization process, inconsistent randomization process for different indications |
| Allocation concealment | Unclear | - No information provided |
| Blinding of participants, personnel and outcome assessors | Unclear | - Blinding process only mentioned for psoriasis study |
| Incomplete outcome data | Low | - All patients accounted for |
| Selective outcome reporting | Low | - All of study’s pre-specified outcomes have been reported |
| Other sources of bias | Low | - The study appears to be free of other sources of bias |

Table 1.21: Risk of Bias Assessment for *study TRANSFIGURE (NCT01807520)*

| Domain | Risk of Bias | Comments |
| --- | --- | --- |
| Sequence generation | Unclear | - No further information on randomization system |
| Allocation concealment | Unclear | - No further information |
| Blinding of participants, personnel and outcome assessors | Low | - Patients and investigators were blinded to treatment allocation |
| Incomplete outcome data | Low | - All patients accounted for |
| Selective outcome reporting | Low | - All of study’s pre-specified outcomes have been reported |
| Other sources of bias | Low | - The study appears to be free of other sources of bias |

Table 1.22: Risk of Bias Assessment for study CARIMA (NCT02559622)

| Domain | Risk of Bias | Comments |
| --- | --- | --- |
| Sequence generation | Unclear | - No further information on randomization system |
| Allocation concealment | Unclear | - No further information |
| Blinding of participants, personnel and outcome assessors | Low | - Participant, Care Provider, Investigator, Outcomes Assessor were blinded to treatment allocation |
| Incomplete outcome data | Low | - All patients accounted for |
| Selective outcome reporting | Low | - All of study’s pre-specified outcomes have been reported |
| Other sources of bias | Low | - The study appears to be free of other sources of bias |

Table 1.23: Risk of Bias Assessment for *Sticherling M, 2017 (NCT02474082)*

| Domain | Risk of Bias | Comments |
| --- | --- | --- |
| Sequence generation | Low | - Automated randomization list |
| Allocation concealment | High | - Randomization numbers were assigned to patients by the investigators in consecutive order |
| Blinding of participants, personnel and outcome assessors | Low | - Open label treatment, efficacy parameters were assessed by blinded assessor who were not involved in any other study procedures and who did not have access to the allocation data or case report forms |
| Incomplete outcome data | Low | - All patients accounted for |
| Selective outcome reporting | Low | - All of study’s pre-specified outcomes have been reported |
| Other sources of bias | Low | - The study appears to be free of other sources of bias |

Table 1.24: Risk of Bias Assessment for *Bagel J, 2017 (NCT02267135)*

| Domain | Risk of Bias | Comments |
| --- | --- | --- |
| Sequence generation | Low | - Randomization done using interactive response technology (IRT) |
| Allocation concealment | Low | - Central allocation |
| Blinding of participants, personnel and outcome assessors | Low | - Patients and investigators were blinded to treatment allocation |
| Incomplete outcome data | Low | - All patients accounted for |
| Selective outcome reporting | Low | - All of study’s pre-specified outcomes have been reported |
| Other sources of bias | Low | - The study appears to be free of other sources of bias |

Table 1.25: Risk of Bias Assessment for *Thaci D, 2015 (NCT02074982)*

| Domain | Risk of Bias | Comments |
| --- | --- | --- |
| Sequence generation | Low | - Randomization done using interactive response technology (IRT) |
| Allocation concealment | Low | - Central allocation |
| Blinding of participants, personnel and outcome assessors | Low | - Patients and investigators were blinded to treatment allocation |
| Incomplete outcome data | Low | - All patients accounted for |
| Selective outcome reporting | Low | - All of study’s pre-specified outcomes have been reported |
| Other sources of bias | Low | - The study appears to be free of other sources of bias |

Table 1.26: Risk of Bias Assessment for *Gottlieb A, 2017 (NCT01806597)*

| Domain | Risk of Bias | Comments |
| --- | --- | --- |
| Sequence generation | Low | - Randomization done using interactive response technology (IRT) |
| Allocation concealment | Low | - Central allocation |
| Blinding of participants, personnel and outcome assessors | Low | - Patients and investigators were blinded to treatment allocation |
| Incomplete outcome data | Low | - All patients accounted for |
| Selective outcome reporting | Low | - All of study’s pre-specified outcomes have been reported |
| Other sources of bias | Low | - The study appears to be free of other sources of bias |

Table 1.27: Risk of Bias Assessment for *Baeten D, 2013 (NCT00809159)*

| Domain | Risk of Bias | Comments |
| --- | --- | --- |
| Sequence generation | Low | - Randomization done using validated system |
| Allocation concealment | Low | - Central allocation |
| Blinding of participants, personnel and outcome assessors | Low | - Patients and investigators were blinded to treatment allocation |
| Incomplete outcome data | Low | - All patients accounted for |
| Selective outcome reporting | Low | - All of study’s pre-specified outcomes have been reported |
| Other sources of bias | Low | - The study appears to be free of other sources of bias |

Table 1.28: Risk of Bias Assessment for *Beaten D, 2015 (NCT01358175 and NCT01649375)*

| Domain | Risk of Bias | Comments |
| --- | --- | --- |
| Sequence generation | Low | - Randomization done using validated system |
| Allocation concealment | Low | - Central allocation |
| Blinding of participants, personnel and outcome assessors | Low | - Patients and investigators were blinded to treatment allocation |
| Incomplete outcome data | Low | - All patients accounted for |
| Selective outcome reporting | Low | - All of study’s pre-specified outcomes have been reported |
| Other sources of bias | Low | - The study appears to be free of other sources of bias |

Table 1.29: Risk of Bias Assessment for *Braun J, 2016 (NCT01358175)*

| Domain | Risk of Bias | Comments |
| --- | --- | --- |
| Sequence generation | Low | - Randomization and Rerandomization done using validated system |
| Allocation concealment | Low | - Central allocation |
| Blinding of participants, personnel and outcome assessors | Low | - Patients and investigators were blinded to treatment allocation, scoring was conducted by two central, independent readers |
| Incomplete outcome data | Low | - All patients accounted for |
| Selective outcome reporting | Low | - All of study’s pre-specified outcomes have been reported |
| Other sources of bias | Low | - The study appears to be free of other sources of bias |

Table 1.30: Risk of Bias Assessment for *Marzo-Ortega H, 2017 (NCT01649375)*

| Domain | Risk of Bias | Comments |
| --- | --- | --- |
| Sequence generation | Low | - Randomization and Rerandomization done using validated system |
| Allocation concealment | Low | - Central allocation |
| Blinding of participants, personnel and outcome assessors | Low | - Patients and investigators were blinded to treatment allocation, all patients received secukinumab from week 16 onward |
| Incomplete outcome data | Low | - All patients accounted for |
| Selective outcome reporting | Low | - All of study’s pre-specified outcomes have been reported |
| Other sources of bias | Low | - The study appears to be free of other sources of bias |

Table 1.31: Risk of Bias Assessment for *Marzo-Ortega H, 2017 (NCT01649375)*

| Domain | Risk of Bias | Comments |
| --- | --- | --- |
| Sequence generation | Low | - Randomization and Rerandomization done using validated system |
| Allocation concealment | Low | - Central allocation |
| Blinding of participants, personnel and outcome assessors | High | - Patients and investigators were blinded to treatment allocation, unblinding occurred after the week 52 analysis |
| Incomplete outcome data | Low | - All patients accounted for |
| Selective outcome reporting | Low | - All of study’s pre-specified outcomes have been reported |
| Other sources of bias | Low | - The study appears to be free of other sources of bias |

Table 1.32: Risk of Bias Assessment for Pavelka K, *2017 (NCT02008916)*

| Domain | Risk of Bias | Comments |
| --- | --- | --- |
| Sequence generation | Low | - Randomization done using interactive response technology (IRT) |
| Allocation concealment | Low | - Central allocation |
| Blinding of participants, personnel and outcome assessors | Low | - Double-blinding |
| Incomplete outcome data | Low | - All patients accounted for |
| Selective outcome reporting | Low | - All of study’s pre-specified outcomes have been reported |
| Other sources of bias | Low | - The study appears to be free of other sources of bias |

Table 1.33: Risk of Bias Assessment for *Kivitz AJ, 2018 (NCT02159053)*

| Domain | Risk of Bias | Comments |
| --- | --- | --- |
| Sequence generation | Low | - Randomization done using interactive response technology (IRT) |
| Allocation concealment | Low | - Central allocation |
| Blinding of participants, personnel and outcome assessors | Low | - Double-blinding |
| Incomplete outcome data | Low | - All patients accounted for |
| Selective outcome reporting | Low | - All of study’s pre-specified outcomes have been reported |
| Other sources of bias | Low | - The study appears to be free of other sources of bias |

Table 1.34: Risk of Bias Assessment for Genovese MC, 2013 *(NCT00928512)*

| Domain | Risk of Bias | Comments |
| --- | --- | --- |
| Sequence generation | Low | - Randomization done using validated system |
| Allocation concealment | Low | - Central allocation |
| Blinding of participants, personnel and outcome assessors | Low | - Double-blinding, no specific information |
| Incomplete outcome data | Low | - All patients accounted for |
| Selective outcome reporting | Low | - All of study’s pre-specified outcomes have been reported |
| Other sources of bias | Low | - The study appears to be free of other sources of bias |

Table 1.35: Risk of Bias Assessment for *Genovese MC, 2014 (NCT00928512)*

| Domain | Risk of Bias | Comments |
| --- | --- | --- |
| Sequence generation | Low | - Rerandomization done using validated system |
| Allocation concealment | Low | - Central allocation |
| Blinding of participants, personnel and outcome assessors | Low | - Double-blinding, no specific information |
| Incomplete outcome data | Low | - All patients accounted for |
| Selective outcome reporting | Low | - All of study’s pre-specified outcomes have been reported |
| Other sources of bias | Low | - The study appears to be free of other sources of bias |

Table 1.36: Risk of Bias Assessment for *Tlustochowicz W, 2016 (NCT01359943)*

| Domain | Risk of Bias | Comments |
| --- | --- | --- |
| Sequence generation | Low | - Randomization done using validated system |
| Allocation concealment | Low | - Central allocation |
| Blinding of participants, personnel and outcome assessors | Low | - Double-blinding |
| Incomplete outcome data | Low | - All patients accounted for |
| Selective outcome reporting | Low | - All of study’s pre-specified outcomes have been reported |
| Other sources of bias | Low | - The study appears to be free of other sources of bias |

Table 1.37: Risk of Bias Assessment for *Blanco FJ, 2017 (NCT01350804)*

| Domain | Risk of Bias | Comments |
| --- | --- | --- |
| Sequence generation | Low | - Randomization done using validated system |
| Allocation concealment | Low | - Central allocation |
| Blinding of participants, personnel and outcome assessors | Low | - Double-blinding, rerandomization at week 16 in placebo/active comparator non-responders |
| Incomplete outcome data | Low | - All patients accounted for |
| Selective outcome reporting | Low | - All of study’s pre-specified outcomes have been reported |
| Other sources of bias | Low | - The study appears to be free of other sources of bias |

Table 1.38: Risk of Bias Assessment for Tahir H, 2017 *(NCT01377012)*

| Domain | Risk of Bias | Comments |
| --- | --- | --- |
| Sequence generation | Low | - Randomization and Rerandomization done using validated system |
| Allocation concealment | Low | - Central allocation |
| Blinding of participants, personnel and outcome assessors | Low | - Double-blinding, rerandomization in placebo nonresponders |
| Incomplete outcome data | Low | - All patients accounted for |
| Selective outcome reporting | Low | - All of study’s pre-specified outcomes have been reported |
| Other sources of bias | Low | - The study appears to be free of other sources of bias |

Table 1.39: Risk of Bias Assessment for *Leonardi C, 2012 (NCT01107457)*

| Domain | Risk of Bias | Comments |
| --- | --- | --- |
| Sequence generation | Low | - Randomization done using validated system |
| Allocation concealment | Low | - Central allocation |
| Blinding of participants, personnel and outcome assessors | Low | - Double-blinding |
| Incomplete outcome data | Low | - All patients accounted for |
| Selective outcome reporting | Low | - All of study’s pre-specified outcomes have been reported |
| Other sources of bias | Low | - The study appears to be free of other sources of bias |

Table 1.40: Risk of Bias Assessment for Gordon KB, 2016 *(NCT0147451, NCT01597245, NCT01646177)*

| Domain | Risk of Bias | Comments |
| --- | --- | --- |
| Sequence generation | Low | - Randomization done using validated system |
| Allocation concealment | Low | - Central allocation |
| Blinding of participants, personnel and outcome assessors | Low | - At week 12 in the UNCOVER-3 trial patients entered a long-term extension period with open label ixekizumab, at week 12 in the UNCOVER-1 and UNCOVER-2 trials, patients who responded to ixekizumab were rerandomized to ixekizumab or placebo |
| Incomplete outcome data | Low | - All patients accounted for |
| Selective outcome reporting | Low | - All of study’s pre-specified outcomes have been reported |
| Other sources of bias | Low | - The study appears to be free of other sources of bias |

Table 1.41: Risk of Bias Assessment for Langley RG, *2018 (NCT02513550)*

| Domain | Risk of Bias | Comments |
| --- | --- | --- |
| Sequence generation | Low | - Randomization done using validated system |
| Allocation concealment | Low | - Central allocation |
| Blinding of participants, personnel and outcome assessors | Low | - Double-blinded |
| Incomplete outcome data | Low | - All patients accounted for |
| Selective outcome reporting | Low | - All of study’s pre-specified outcomes have been reported |
| Other sources of bias | Low | - The study appears to be free of other sources of bias |

Table 1.42: Risk of Bias Assessment for *Ryan C, 2018 (NCT02718898)*

| Domain | Risk of Bias | Comments |
| --- | --- | --- |
| Sequence generation | Low | - Assignments to treatment groups were performed using a computer-generated random sequence |
| Allocation concealment | Low | - Central allocation |
| Blinding of participants, personnel and outcome assessors | Low | - Double-blinded |
| Incomplete outcome data | Low | - All patients accounted for |
| Selective outcome reporting | Low | - All of study’s pre-specified outcomes have been reported |
| Other sources of bias | Low | - The study appears to be free of other sources of bias |

Table 1.43: Risk of Bias Assessment for *Reich K, 2017 (NCT02561806)*

| Domain | Risk of Bias | Comments |
| --- | --- | --- |
| Sequence generation | Low | - Randomization via interactive web-response system |
| Allocation concealment | Low | - Central allocation |
| Blinding of participants, personnel and outcome assessors | Low | - Double-blinded |
| Incomplete outcome data | Low | - All patients accounted for |
| Selective outcome reporting | Low | - All of study’s pre-specified outcomes have been reported |
| Other sources of bias | Low | - The study appears to be free of other sources of bias |

Table 1.44: Risk of Bias Assessment for *Mease PJ, 2017 (NCT01695239)*

| Domain | Risk of Bias | Comments |
| --- | --- | --- |
| Sequence generation | Low | - Randomization via an interactive voice response system based on a computer-generated randomization code |
| Allocation concealment | Low | - Central allocation |
| Blinding of participants, personnel and outcome assessors | Low | - Double-blinded |
| Incomplete outcome data | Low | - All patients accounted for |
| Selective outcome reporting | Low | - All of study’s pre-specified outcomes have been reported |
| Other sources of bias | Low | - The study appears to be free of other sources of bias |

Table 1.45: Risk of Bias Assessment for *Nash P, 2017 (NCT02349295)*

| Domain | Risk of Bias | Comments |
| --- | --- | --- |
| Sequence generation | Low | - Assignments to treatment groups were performed using a computer-generated random sequence |
| Allocation concealment | Low | - Central allocation |
| Blinding of participants, personnel and outcome assessors | Low | - Double-blinded |
| Incomplete outcome data | Low | - All patients accounted for |
| Selective outcome reporting | Low | - All of study’s pre-specified outcomes have been reported |
| Other sources of bias | Low | - The study appears to be free of other sources of bias |

Table 1.46: Risk of Bias Assessment for *van der Hejde D, 2018 (NCT01695239)*

| Domain | Risk of Bias | Comments |
| --- | --- | --- |
| Sequence generation | Low | - Randomization done using validated system |
| Allocation concealment | Low | - Central allocation |
| Blinding of participants, personnel and outcome assessors | Low | - Double-blinded |
| Incomplete outcome data | Low | - All patients accounted for |
| Selective outcome reporting | Low | - All of study’s pre-specified outcomes have been reported |
| Other sources of bias | Low | - The study appears to be free of other sources of bias |

Table 1.47: Risk of Bias Assessment for *Genovese MC, 2010*

| Domain | Risk of Bias | Comments |
| --- | --- | --- |
| Sequence generation | Unclear | - No information on part A, part B used an interactive voice response system for randomization |
| Allocation concealment | High | - Randomization during part A was performed manually by a study drug coordinator, and part B used an interactive voice response system for randomization |
| Blinding of participants, personnel and outcome assessors | Low | - Double-blinded |
| Incomplete outcome data | Low | - All patients accounted for |
| Selective outcome reporting | Low | - All of study’s pre-specified outcomes have been reported |
| Other sources of bias | Low | - The study appears to be free of other sources of bias |

Table 1.48: Risk of Bias Assessment for *Genovese MC, 2014 (NCT00966875)*

| Domain | Risk of Bias | Comments |
| --- | --- | --- |
| Sequence generation | Low | - Randomization by computer-generated random sequence using an interactive voice response system |
| Allocation concealment | Low | - Central allocation |
| Blinding of participants, personnel and outcome assessors | Low | - Double-blinded |
| Incomplete outcome data | Low | - All patients accounted for |
| Selective outcome reporting | Low | - All of study’s pre-specified outcomes have been reported |
| Other sources of bias | Low | - The study appears to be free of other sources of bias |

Table 1.49: Risk of Bias Assessment for *Genovese MC, 2016 (NCT00966875)*

| Domain | Risk of Bias | Comments |
| --- | --- | --- |
| Sequence generation | Low | - Randomization done using validated system |
| Allocation concealment | Low | - Central allocation |
| Blinding of participants, personnel and outcome assessors | Low | - Double-blinded |
| Incomplete outcome data | Low | - All patients accounted for |
| Selective outcome reporting | Low | - All of study’s pre-specified outcomes have been reported |
| Other sources of bias | Low | - The study appears to be free of other sources of bias |

Table 1.50: Risk of Bias Assessment for *Papp KA, 2012 (NCT00975637)*

| Domain | Risk of Bias | Comments |
| --- | --- | --- |
| Sequence generation | Low | - Randomization done using validated system |
| Allocation concealment | Low | - Central allocation |
| Blinding of participants, personnel and outcome assessors | Low | - Double-blinded |
| Incomplete outcome data | Low | - All patients accounted for |
| Selective outcome reporting | Low | - All of study’s pre-specified outcomes have been reported |
| Other sources of bias | Low | - The study appears to be free of other sources of bias |

Table 1.51: Risk of Bias Assessment for *Nakagawa H, 2015 (NCT01748539)*

| Domain | Risk of Bias | Comments |
| --- | --- | --- |
| Sequence generation | Low | - Randomization done using validated system |
| Allocation concealment | Low | - Central allocation |
| Blinding of participants, personnel and outcome assessors | Low | - Double-blinded |
| Incomplete outcome data | Low | - All patients accounted for |
| Selective outcome reporting | Low | - All of study’s pre-specified outcomes have been reported |
| Other sources of bias | Low | - The study appears to be free of other sources of bias |

Table 1.52: Risk of Bias Assessment for *Papp KA, 2016 (NCT01708590)*

| Domain | Risk of Bias | Comments |
| --- | --- | --- |
| Sequence generation | Low | - Randomization via an interactive voice response system |
| Allocation concealment | Low | - Central allocation |
| Blinding of participants, personnel and outcome assessors | Low | - Double-blinded |
| Incomplete outcome data | Low | - All patients accounted for |
| Selective outcome reporting | Low | - All of study’s pre-specified outcomes have been reported |
| Other sources of bias | Low | - The study appears to be free of other sources of bias |

Table 1.53: Risk of Bias Assessment for *Lebwohl M, 2015 (NCT01708603, NCT01708629)*

| Domain | Risk of Bias | Comments |
| --- | --- | --- |
| Sequence generation | Low | - Randomization lists were generated with the use of a permuted block design |
| Allocation concealment | Low | - Central allocation |
| Blinding of participants, personnel and outcome assessors | Low | - Double-blinded |
| Incomplete outcome data | Low | - All patients accounted for |
| Selective outcome reporting | Low | - All of study’s pre-specified outcomes have been reported |
| Other sources of bias | Low | - The study appears to be free of other sources of bias |

Table 1.54: Risk of Bias Assessment for *Mease PJ, 2014 (NCT01516957)*

| Domain | Risk of Bias | Comments |
| --- | --- | --- |
| Sequence generation | Low | - Randomization lists were generated by Amgen representatives with the use of a permutted-block design |
| Allocation concealment | Low | - Randomization lists were generated by Amgen representatives with the use of a permutted-block design |
| Blinding of participants, personnel and outcome assessors | Low | - Double-blinded |
| Incomplete outcome data | Low | - All patients accounted for |
| Selective outcome reporting | Low | - All of study’s pre-specified outcomes have been reported |
| Other sources of bias | Low | - The study appears to be free of other sources of bias |

Table 1.55: Risk of Bias Assessment for *Martin DA, 2013 (NCT00771030)*

| Domain | Risk of Bias | Comments |
| --- | --- | --- |
| Sequence generation | Low | - Randomization done using validated system |
| Allocation concealment | Low | - Central allocation |
| Blinding of participants, personnel and outcome assessors | Low | - Double-blinded |
| Incomplete outcome data | Low | - All patients accounted for |
| Selective outcome reporting | Low | - All of study’s pre-specified outcomes have been reported |
| Other sources of bias | Low | - The study appears to be free of other sources of bias |

Table 1.56: Risk of Bias Assessment for *Pavelka K, 2015 (NCT00950989)*

| Domain | Risk of Bias | Comments |
| --- | --- | --- |
| Sequence generation | Low | - Randomization done using validated system |
| Allocation concealment | Low | - Central allocation |
| Blinding of participants, personnel and outcome assessors | Low | - Double-blinded |
| Incomplete outcome data | Low | - All patients accounted for |
| Selective outcome reporting | Low | - All of study’s pre-specified outcomes have been reported |
| Other sources of bias | Low | - The study appears to be free of other sources of bias |

# **Newcastle-Ottawa assessment for non randomized trials**

2.1: Study JP01 (NCT02547714)

*Selection*

1. Representation of the exposed cohort (a) *
2. No non exposed cohort, single arm trials (c)
3. Ascertainment of exposure (a)*
4. Demonstration that outcomes of interest was not present at start of study (b)

*Comparability*

1. No cohort for comparison

*Outcome*

1. Assessment of Outcome (a)*
2. Was follow-up long enough for outcomes to occur (a)*
3. Adequacy of follow up of cohorts (b)*

Total Newcastle-Ottawa score: 5

2.2: Imafuku S, 2016 (NCT01952015)

*Selection*

1. Representation of the exposed cohort (a) *
2. No non exposed cohort, single arm trials (c)
3. Ascertainment of exposure (a)*
4. Demonstration that outcomes of interest was not present at start of study (b)

*Comparability*

1. No cohort for comparison

*Outcome*

1. Assessment of Outcome (a)*
2. Was follow-up long enough for outcomes to occur (a)*
3. Adequacy of follow up of cohorts (b)*

Total Newcastle-Ottawa score: 5

2.3: Baraliakos X, 2018 (NCT01863732)

*Selection*

1. Representation of the exposed cohort (a) *
2. Selection of the non-exposed cohort (a)*
3. Ascertainment of exposure (a)*
4. Demonstration that outcomes of interest was not present at start of study (b)

*Comparability*

1. Comparability of cohorts on the basis of the design or analysis – yes – age, previous therapy **

*Outcome*

1. Assessment of Outcome (a ) *
2. Was follow-up long enough for outcomes to occur (a)*
3. Adequacy of follow up of cohorts (b)*

Total Newcastle-Ottawa score: 8

2.4: Gordon KB, 2014 (NCT01107457)

*Selection*

1. Representation of the exposed cohort (a) *
2. No non exposed cohort, single arm trials (c)
3. Ascertainment of exposure (a)*
4. Demonstration that outcomes of interest was not present at start of study (b)

*Comparability*

1. No cohort for comparison

*Outcome*

1. Assessment of Outcome (a)*
2. Was follow-up long enough for outcomes to occur (a)*
3. Adequacy of follow up of cohorts (b)*

Total Newcastle-Ottawa score: 5

2.5: Blauvelt A, 2017 (NCT01646177)

*Selection*

1. Representation of the exposed cohort (a) *
2. No non exposed cohort, single arm trials (c)
3. Ascertainment of exposure (a)*
4. Demonstration that outcomes of interest was not present at start of study (b)

*Comparability*

1. No cohort for comparison

*Outcome*

1. Assessment of Outcome (a)*
2. Was follow-up long enough for outcomes to occur (a)*
3. Adequacy of follow up of cohorts (b)*

Total Newcastle-Ottawa score: 5

2.6: Leonardi C, 2018 (NCT01646177)

*Selection*

1. Representation of the exposed cohort (a) *
2. No non exposed cohort, single arm trials (c)
3. Ascertainment of exposure (a)*
4. Demonstration that outcomes of interest was not present at start of study (b)

*Comparability*

1. No cohort for comparison

*Outcome*

1. Assessment of Outcome (a)*
2. Was follow-up long enough for outcomes to occur (a)*
3. Adequacy of follow up of cohorts (b)*

Total Newcastle-Ottawa score: 5

2.7: Saeki H, 2015 (NCT01624233)

*Selection*

1. Representation of the exposed cohort (a) *
2. No non exposed cohort, single arm trials (c)
3. Ascertainment of exposure (a)*
4. Demonstration that outcomes of interest was not present at start of study (b)

*Comparability*

1. No cohort for comparison

*Outcome*

1. Assessment of Outcome (a)*
2. Was follow-up long enough for outcomes to occur (a)*
3. Adequacy of follow up of cohorts (b)*

Total Newcastle-Ottawa score: 5

2.8: Saeki H, 2017 (NCT01624233)

*Selection*

1. Representation of the exposed cohort (a) *
2. No non exposed cohort, single arm trials (c)
3. Ascertainment of exposure (a)*
4. Demonstration that outcomes of interest was not present at start of study (b)

*Comparability*

1. No cohort for comparison

*Outcome*

1. Assessment of Outcome (a)*
2. Was follow-up long enough for outcomes to occur (a)*
3. Adequacy of follow up of cohorts (b)*

Total Newcastle-Ottawa score: 5

2.9: Papp KA, 2014 (NCT01101100)

*Selection*

1. Representation of the exposed cohort (a) *
2. No non exposed cohort, single arm trials (c)
3. Ascertainment of exposure (a)*
4. Demonstration that outcomes of interest was not present at start of study (b)

*Comparability*

1. No cohort for comparison

*Outcome*

1. Assessment of Outcome (a)*
2. Was follow-up long enough for outcomes to occur (a)*
3. Adequacy of follow up of cohorts (b)*

Total Newcastle-Ottawa score: 5

2.10: Yamasaki K, 2016 (NCT01782937)

*Selection*

1. Representation of the exposed cohort (a) *
2. No non exposed cohort, single arm trials (c)
3. Ascertainment of exposure (a)*
4. Demonstration that outcomes of interest was not present at start of study (b)

*Comparability*

1. No cohort for comparison

*Outcome*

1. Assessment of Outcome (a)*
2. Was follow-up long enough for outcomes to occur (a)*
3. Adequacy of follow up of cohorts (b)*

Total Newcastle-Ottawa score: 5

# **Overall quality assessment, on basis of the Grading of Recommendations Assessment, Development, and Evaluation (GRADE) criteria**

| Study | Starting Level of Evidence | Reasons for decreasing the level of evidence | | | | | Reasons to increase level of evidence (strong association, plausible confounding and bias adjustment) | Final level of evidence |
| --- | --- | --- | --- | --- | --- | --- | --- | --- |
|  |  | Risk of bias | Inconsistency | Indirectness | Imprecision | Publication bias |  |  |
| Randomized trials - 56 | High | ↔ | ↔ | ↔ | ↓ | ↔ | ↔ | Moderate |
| Observational trials - 10 | Low | ↔ | ↔ | ↔ | ↓ | ↔ | ↔ | Very low |
